# Design and validation of a novel multiple sites signal acquisition and analysis system based on pressure stimulation for human cardiovascular information

**DOI:** 10.1038/s41598-025-97812-8

**Published:** 2025-04-18

**Authors:** Gaiqin Liu, Yuan Li, Longcong Chen, Juan Jiang, Jie Tian, Panpan Feng

**Affiliations:** 1 Medical Information College, Chongqing Medical University, Chongqing, People’s Republic of China; 2 Department of Cardiology, Children’s Hospital of Chongqing Medical University, Chongqing, People’s Republic of China; 3 Department of Cardiology, The First Affiliated Hospital of Chongqing Medical University, Chongqing, People’s Republic of China; 4 School of science, Chongqing University of Technology, Chongqing, People’s Republic of China; 5 Department of Geriatrics, Chongqing Mental Health Center, Chongqing, People’s Republic of China

**Keywords:** Cardiovascular information, Electrocardiogram (ECG), Heart sound signal, Lung sound signal, Pressure stimulation, Pulse signal, Biomedical engineering, Design, synthesis and processing, Data acquisition, Population screening, Physical examination

## Abstract

Cardiovascular diseases (CVDs) pose a significant threat to human health and place considerable strain on healthcare systems. Therefore, it is crucial to maximize the acquisition of cardiovascular information (CVI) through non-invasive methods to enhance early screening, diagnosis, and evaluation of CVDs. Numerous studies have demonstrated that obtaining more CVI by simultaneously acquiring multi-site signals and applying pressure stimulation at specific sites, such as blood pressure measurement, is an effective approach. Based on this evidence, we proposed a novel signal acquisition-and-analysis system to gather comprehensive CVI through a combination of a non-pressure and six pressure-stimulation sub-processes. This system involves the novelty of applying slowly gradual decrease, personalized maximum-pulse amplitude, and blocking blood-flow pressure to six cuffs placed on both arms, wrists, and ankles in a predetermined time sequence. During each sub-process, the system has newly integrated the multi-site simultaneous collection of 27-channel non-invasive signals, including electrocardiogram, heart sound, lung sound, photoplethysmographic-and-pressure pulse. To ensure measurement accuracy, three types of verification-and-calibration instruments were employed. Our results demonstrate that the system can achieve simultaneous acquisition of 27-channel signals during each sub-process, yielding both novel and traditional cardiovascular parameters with high accuracy and good stability. Furthermore, the results suggest that the system can facilitate in-depth research into the relationships between collected signals and CVDs, provide rich raw data for cardiovascular health assessment and disease prediction models based on machine learning algorithms, and offer a new non-invasive method for early diagnosis, evaluation, and prediction of CVDs.

**Supplementary Information:**

The online version contains supplementary material available at 10.1038/s41598-025-97812-8.

## Introduction

Cardiovascular diseases (CVDs, see Supplementary Table S1 for all abbreviation and their full names in this paper) are the leading cause of mortality worldwide, impairing vital organ function and potentially resulting in severe conditions such as heart attack or stroke^1^. As reported by the World Health Organization, they account for 32% of all global fatalities, with an estimated annual death toll of 17.9 million. Heart attacks are responsible for over 85% of all cardiovascular-related deaths^2^. The developmental stages of CVDs can be categorized into early (or low-risk) stage, sub-clinical stage, and stage characterized by frequent cardiovascular events. Each stage exhibits varying patient numbers and disease presentations. However, overall risk gradually increases with age and lifestyle factors^3^. In the early stages of the disease, patients typically exhibit superior physical condition and greater treatment tolerance, resulting in improved therapeutic outcomes. Moreover, timely intervention can effectively mitigate complications, alleviate patient suffering, and alleviate financial burdens^4–7^. Therefore, the early diagnosis of CVDs holds immense significance in mitigating the incidence of cardiovascular events, enhancing patients’ quality of life, and reducing healthcare expenditures.

To date, numerous studies have already verified that many cardiovascular parameters (CVPs) can be effectually utilized for early diagnosis and assessment of CVDs^8^. When some CVDs, including arteriosclerosis, hypertension, and coronary heart disease, are in the early stages, they often lack self-conscious symptoms. However, certain CVPs, such as blood pressure, blood vessel elasticity, heart rate, and pulse wave velocity (PWV), undergo significant changes. Therefore, many researchers have directed their attention towards extracting more CVPs with clinical value by non-invasive methods^9–12^. Although numerous CVPs can be obtained using available instruments and equipment in hospitals like duplex ultrasonography, X-ray computed tomography, and arteriosclerosis measuring instruments, these traditional methods require professional operation and relatively high costs, which render them unsuitable for extensive screening and frequent testing. To address their limitations, numerous studies have employed non-invasive and easily obtainable physiological signals to extract various CVPs^13,14^. The physiological signals encompass electrocardiogram (ECG), photoplethysmography (PPG)^15^, pressure pulse, heart sound (HS), and others^16–19^. Many studies have demonstrated that each signal alone can yield several clinically significant CVPs. From the electrocardiogram (ECG), many CVPs, including R-R wave interval, QRS complex characteristics, P wave morphology, and S-T segment changes, can be derived. These parameters reflect both the electrophysiological properties of the heart (such as premature beats or arrhythmias) and structural abnormalities (like ventricular hypertrophy and atrial enlargement)^20–24^. Additionally, the CVPs related to HS, such as the relative intensity and duration of the first sound (S1) and second heart sound (S2) can be obtained through analysis of the HS signal, and can be applied to evaluate the stenosis, insufficiency, functional abnormalities of heart valve, myocardial contractility, and ventricular filling status^25,26^. Furthermore, multi-channel signal acquisition and analysis are researched to enhance the extraction of cardiovascular parameters (CVPs). For instance, by integrating ECG and PPG signals, it becomes possible to obtain PWV, a measure that assesses arterial stiffness^27^, and improve the accuracy of non-invasive blood pressure measurement with cuff pressure help^28,29^. A lot of studies have consistently demonstrated that the simultaneous collection of cardiovascular-related signals from multiple sites on the human body leads to an increased number of CVPs and enables a more accurate assessment of cardiovascular health^30–35^, which is the first basic idea of this paper.

In medical research, it has proven to be an effective approach for obtaining comprehensive information about the human body by applying various incentives or stimuli to the body and detecting its response. For example, in brain science research, evoked potentials are collected during visual or auditory stimulation by electroencephalography. Evoked potentials can provide more extensive insights into the human brain compared to background electroencephalogram^36,37^. The cuff-based oscillometric method that uses pressure stimuli is an effective way of blood pressure measurement, which provides a method for the monitoring of CVDs^38^. Similarly, it is evident that a greater amount of cardiovascular information (CVI), including CVPs, can be extracted from the collected cardiovascular signals at different sites when specific interrelated stimuli are applied to the human body. This concept forms the second basic idea of this paper. Specifically, we propose a pressure stimulation-based system to enhance the acquisition of CVI. By applying different pressures to cuffs placed on the arms, wrists, and ankles, distinct deformations in blood vessels at corresponding positions can be induced, which will lead to changes in both blood flow dynamics and pulse wave characteristics at the fingers and toes. Therefore, by analyzing these variations, it is expected to obtain more valuable CVPs. In this study, our designed system incorporates three types of pressure: slowly decreasing pressure, maximum pulse amplitude pressure (MPAP), and blocking blood flow pressure (BBFP). Furthermore, considering that the human body is an integrated whole, and the signals at its various sites are necessarily related, analyzing their relationships should enable us to obtain additional clinically relevant CVPs. Additionally, the ECG and HS signals contain vital information about cardiac health while the lung sound (LS) signal reflects blood oxygen exchange and respiration in some extent. Finally, based on the aforementioned two basic ideas, we propose a novel multi-channel and multi-site signal acquisition and analysis system based on pressure stimulation for human cardiovascular information in this paper. This system enables synchronous collection of 27-channel signals, including photoelectric pulse at bilateral earlobes, fingers, and toes, pressure and pressure pulse waves at bilateral arms, wrists, and ankles, as well as single-channel ECG, HS, and LS. In addition, for achieving more accurate and comprehensive CVPs, the entire acquisition process is divided into seven sub-processes according to different pressure stimulation modes implemented within the system. Overall, our proposed system is expected to serve as an effective tool for in-depth cardiovascular studies encompassing comprehensive examination and evaluation of cardiovascular function along with an analysis module dedicated to synchronization signals related to the cardiovascular domain. Furthermore, it can provide abundant raw data for machine learning-based assessment of cardiovascular health and prediction models for CVDs, such as data of HS^39–43^, ECG^22,40,44–48^, LS^49^, and PPG^13,18,50^. Moreover, this research lays a solid foundation for developing more comprehensive, effective, non-invasive, safe, and reliable testing systems/devices specifically designed for assessing various aspects of cardiovascular function that could be utilized both in hospital settings or even at home.

## System structure

**Fig. 1:**
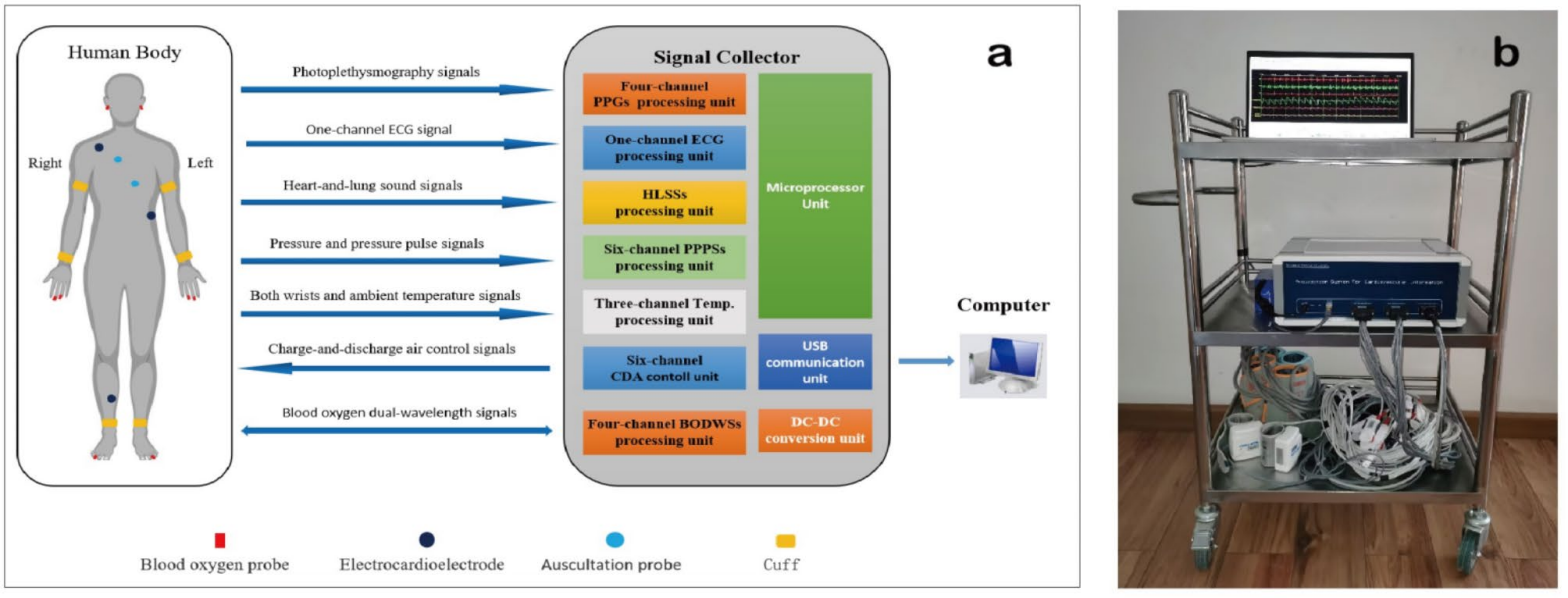
Diagram of the system. (**a**) block diagram; (**b**) real picture.

On the whole, the system consists of hardware and software, with its structural block diagram displayed in Fig. 1(a). The hardware is composed of sensors, a signal collector, and a computer. The software consists of two independent programs, one for the microprocessor of the signal collector and the other for the computer. Detailed descriptions of the hardware and software are provided below.

### Hardware

As illustrated in Fig. 1, the hardware of the system is made up of sensors, a signal collector, and a computer. The sensors are utilized to extract various signals related to CVI from the human body. The signal collector comprises ten units, and realizes the signal processing, various controls, and information exchange with computer via universal serial bus (USB) interface. Combined with our designed software, the computer performs display, storage, and preliminary analysis of various related data and information. The computer used in this system can be any general-purpose computer, such as a personal computer, or notebook computer, so its further elaboration is unnecessary. Therefore, only the sensors and the collector are described below.

#### Sensors

In this system, five types of sensors, including sound, ECG, photo-electricity, pressure, and temperature transducers, are applied. Together with the medical auscultation probes and hoses, the selected sound sensors for detecting heart and lung sounds are the CZ-034As electret microphones manufactured by Panasonic Corporation of Japan. These microphones possess highly sensitive and flat frequency response characteristics. For ECG measurements, disposable ECG electrodes produced by Shanghai Shenfeng Medical & Health Product Corporation Limited in China were utilized. The photoelectric sensors integrated into the blood oxygen probe (YA001), supplied by Shenzhen Yongkangda Technology Corporation Limited in China, were used to acquire PPG and blood-oxygen double wavelength signals (BODWSs). Pressure sensors (MPS-3117-006GCs) made by Metrodyne Microsystem Corporation in the Taiwan province of China were employed to measure the pressure exerted on six cuffs placed on both arms, wrists, and ankles respectively. To obtain the temperature of conditions and both wrists, negative-temperature coefficient thermistor MF52B with ± 1% accuracy ranging from − 40 °C to + 125 °C was applied.

#### Signal collector

The Signal collector of this system consists of a signal processing circuit, metal housing and interface. The signal processing circuit (see Supplementary Fig. S1 for its real picture) is made up of ten elements, including four-channel PPG signals (PPGSs), one-channel ECG signal, two-channel heart and lung sound signals (HLSSs), six-channel pressure and pressure pulse signals (PPPSs), four-channel BODWSs, and three-channel Temp. processing units, and six-channel charge-and-discharge air (CDA) control, microprocessor, USB and direct current to direct current (DC-DC) conversion units.

Each-channel signal processing unit has a same circuit structure, comprising pre-amplification, active filter, and voltage amplification circuits. And its output is fed into the programmable gain amplifier with a multiplexer of the microprocessor unit. Among the ten units, a total of five signal processing units are dedicated to handling 27-channel signals. The pre-amplification circuits for four-channel BODWSs and four-channel PPGs consist of high precision operational amplifiers AD795, while other signals employ high accuracy instrumentation amplifier AD620. They are designed to amplify sensor output signals and provide sufficient amplitude for subsequent active filter circuits. The active filter circuits, tailored to meet the frequency requirements of each channel signal, exhibit some variations across channels. Specifically, each active filter circuit for four-channel BODWSs comprises a 50 Hz trap filter and a second-order low pass filter, and that for six-channel PPPSs includes a 50 Hz trap filter and a second-order low pass filter, whereas each active filter circuit for other 13-channel signals includes a second-order high pass filter, a 50 Hz trap filter, and a second-order low pass filter. All 50 Hz trap filters utilize quad operational amplifiers OPA4188 with low offset voltage (< 25 µV maximum) as well as near zero-drift over time and temperature characteristics. Dual operational amplifiers OPA2188, which has the same performance as OPA4188, are employed in all second-order low pass and second-order high pass filter circuits for each channel signal. Table 1 provides details on hardware-defined frequency ranges for individual signals along with their corresponding sampling frequencies. Additionally, it also displays the number of channels associated with each type of signal.

**Table 1:** Signal type and corresponding parameters.

Signal Type	PPGSs	ECG	HLSSs	PPSs	Pressure signals	BODWSs
Signal-channel Number	4	1	2	6	6	8
Frequency Range (Hz)	0.5–100	0.05–100	20−1000	0.5–100	0−1.5	0–32 K
Sampling rate of Each-channel signal ( s^− 1^)	400	1600	3200	400	400	400

Specifically, it should be noted that the BODWSs are collected alternately at wavelengths of 940 and 660 nm. To mitigate the impact of ambient light, each sample data for BODWSs equals the difference between corresponding-wavelength light irradiation and no-light irradiation. Additionally, in this study, the four-channel BODWSs are collected from the toes and fingers. Since blood oxygen saturation at the fingers and toes changes when pressure stimulation is applied, we hope to obtain more cardiovascular information through these changes. The four-channel PPG signals are acquired at both earlobes and fingers. The PPG signals at the fingers aim to obtain the detailed correlation and discrepancies associated with BODWSs at the fingers. The PPG signals from the earlobes are intended to capture pulse information at those locations. The reason for not employing BODWSs at the earlobes in this study is that the pressure stimulation applied in this study theoretically should not affect the blood oxygen saturation at the earlobes.

The microprocessor unit mainly consists of eleven programmable gain amplifiers with multiplexers, a high-precision reference voltage chip ADR363B, a serial static random access memory chip VTI7064MSME, and a microprocessor STM32F407VGT6. The programmable gain amplifiers with multiplexers include eight PGA112s for HLSSs, BODWSs, and PPGSs, and three PGA116s for ECG and PPPSs. Both the PGA112 and PGA116 devices are zero-drift programmable gain amplifiers that supply software-configured selectable gains of 1, 2, 5, 10, 20, 50, 100 or 200 for different subjects. Moreover, the PGA116 is integrated into a multiplexer with ten analog inputs while the PGA112 has only two analog inputs. To enhance accuracy and stability in signal acquisition processes, a ADR363B is applied in the unit, which offers precision 3.0 V band gap voltage reference with a low-temperature drift of 9 ppm/°C and ± 4 mV accuracy for analog-digital converters integrated into the microprocessor. Additionally, the VTI7064MSME chip, with a capacity of 64 Mbit, is used to temporarily store collected data in case of transmission loss. The microprocessor STM32F407VGT6 incorporates an advanced ARM^®^ Cortex^®^-M4 RISC core operating at a frequency of 168 MHz. It also boasts high-speed embedded memories, including a 1024 Kbytes flash memory, and offers three 12-bit analog-to-digital converters. These converters enable the conversion of various signals from analog to digital, and facilitate diverse control functions through its specialized software.

The six-channel CDA control unit, which comprises three ISO7240Cs and a 74HC573D, is used to manage six cuffs by controlling six air pumps and six deflation valves. The ISO7240C, a quad-channel digital isolator, is employed to mitigate signal acquisition interference caused by the operation of the air pump and vent valve. The 74HC573D serves as an 8-bit D-type transparent latch to drive subsequent circuits. The USB communication unit primarily consists of a CH340E and an ADUM1201C. CH340, a USB conversion chip manufactured by Jiangsu Qinheng Co., Ltd., enables the conversion from USB to a universal asynchronous receiver/transmitter interface, allowing direct communication with the microprocessor without the need for custom USB driver development on the Windows platform. To ensure signal acquisition and transmission integrity while minimizing interference, a dual-channel digital isolator ADUM1201C is placed between CH340 and STM32F407VGT6. The DC-DC conversion unit is responsible for converting the external input direct current voltage (ranging from 8 to 14 V) into various digital and analog power supplies for other units. Additionally, this system includes a three-channel Temp. processing unit for environmental monitoring and wrists temperature sensing alongside an input voltage monitor circuit.

### Software

The system software comprises our designed microprocessor and computer application programs. The microprocessor software, compiled using STM32CubeIDE 1.9.0 and integrated with hardware, accomplishes signal acquisition and control functions such as adjusting the gain of each channel signal, collecting signals in a defined sequence, exchanging information with the computer, and storing set parameters in the microprocessor’s flash memory. It should be noted that data acquisition for each signal in this system is achieved through the direct memory access function of the 12-bit analog-digital converters in the microprocessor. Each acquisition value is obtained by summing four adjacent 12-bit analog-digital conversion values of the corresponding channel signal. Thus, each acquisition value is a 14-bit binary value. Moreover, with a resolution of 0.01mmHg, the output value for each pressure signal is derived by multiplying the 14-digit value with a specific pressure coefficient within the microprocessor. The output values for photo-electric signals at wavelengths of 660 nm and 940 nm are differences between acquisition values under light irradiation and no-light irradiation at fingers and toes, respectively. Additionally, the output values of other signals are the 14-bit binary acquisition value.

The computer-application software is designed to facilitate communication with the microprocessor through the USB interface, as well as enable the display and storage of both each-channel signal data and personal information. Additionally, it allows for the adjustment of various parameters of the signal collector (such as the gain of each-channel signal, the ratio coefficient between the pressure value and analog-digital conversion value (ADCV), deflate speed of each cuff), customization of each-channel signal parameters (including line color and type, order of channel signals, vertical amplitude, horizontal time interval), selection of displayed signal-channel numbers on each screen, and provides time and frequency domain analysis capabilities for specific signals. Furthermore, preliminary analysis results, such as heart rate, blood pressure, mean time between Q-wave and S-wave in ECG signal, mean ratio of amplitude S1 to S2 in HS signal, are also available.

## Signal collecting process

**Fig. 2:**
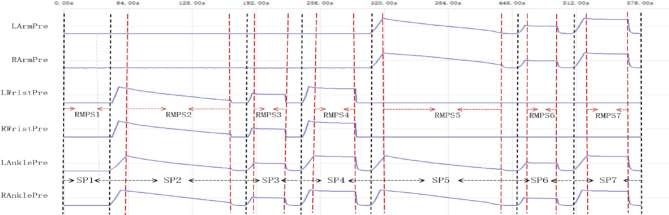
Pressure waveform of six cuffs placed on two-side the arms, wrists, and ankles during the entire signal-collection process.

The entire signal collection is divided into seven sub-processes from SP1 to SP7, as illustrated in Fig. 2. During each sub-process, 27-channel signals are simultaneously acquired and listed in Table 1.

As shown in Fig. 2, the real meaningful procedural segments (RMPSs) of these sub-processes include the entire no-pressure sub-process SP1, the gradual venting procedural segment of SP2, the constant-pressure procedural segment of SP3, the constant-pressure procedural segment of SP4, the gradual venting procedural segment of SP5, the constant-pressure procedural segment of SP6, and constant-pressure procedural segment of SP7, expressed as from RMPS1 to RMPS7 respectively. And there is a non-pressure time interval of about 15 s between each pressure phase. The parameters and purposes of these RMPSs are listed in Table 2. Furthermore, by the computer-application software, the maximum pressure values for RMPS2 and RMPS5, can be set and their default values are 180 mmHg. Additionally, MPAP values for RMPS3 and RMPS6 can be automatically calculated based on their respective preceding segments - namely through RMPS2 for calculating MPAP of RMPS3 and through RMPS5 for MPAP of RMPS6. Moreover, the computer-application software allows for pre-setting the acquisition time of certain segments, such as RMPS1, RMPS3, RMPS4, RMPS6, and RMPS7. Furthermore, the settable parameters can be saved to flash memory integrated within microprocessor by sending specific commands to the collector. Once the related parameters are modified, the system will operate according to new settings.

**Table 2:** The parameters and purpose of RMPSs.

Type	Pressure mode	pressure (mmHg)	pressure cuffs	Approximate time(s)	purpose
RMPS1	non-pressure	0	none	40	Get the signal of each measurement site in its natural state and compare their changes with other processes.
RMPS2	gradual decrease	MP-30	both wrists and ankles	120	Obtain the systolic pressure, diastolic pressure, MPAP, and other information of wrist-and-ankle pressure pulse.
RMPS3	constant	MPAP	both wrists and ankles	30	Obtain pressure pulse information of both two-side wrists and ankles under personalized pressure and relationship with other signals.
RMPS4	constant	MP	both wrists and ankles	40	Obtain information about oxygen consumption and metabolic conditions of fingers and toes when blood flows at the wrists and ankles are blocked.
RMPS5	gradual decrease	MP-30	both arms and ankles	120	Obtain the systolic pressure, diastolic pressure, MPAP, and other information of arm-and-ankle pressure pulse.
RMPS6	constant	MPAP	both arms and ankles	30	Obtain pressure pulse information of both two-side arms and ankles under personalized pressure and relationship with other signals.
RMPS7	constant	MP	both arms and ankles	40	Obtain information about oxygen consumption and metabolic conditions of fingers and toes when blood flows at the arms and ankles are blocked.

MP-maximum pressure; MPAP-maximum pulse amplitude pressure.

## System performance

Based on the frequency requirements of each-channel signal, different sample frequencies, shown in Table 1, are implemented in the system for different types of signals. When starting cardiovascular information collection by this system, it is essential to correctly position the sensors and six cuffs on the appropriate sites and input the subject’s basic information into the computer-application software beforehand. Subsequently, the system will complete the entire signal acquisition process by itself. Throughout this data collection process, real-time waveform displays of all 27-channel signals, shown in Table 1, can be observed simultaneously. It should be noted that six-channel PPPSs correspond to 12-channel signals, (six-channel pressure pulses and six-channel pressure signals). And four-channel BODWSs are eight-channel signals, (four-channel photoelectric signals with the wavelength of 940 nm and four-channel signals with the wavelength of 660 nm). Furthermore, this system enables analysis of all collected 27-channel signals and allows storage of relevant results for subsequent comprehensive research purposes, such as cardiovascular function evaluation or diagnosis/monitoring of CVDs.

### Calibration and validation

**Fig. 3:**
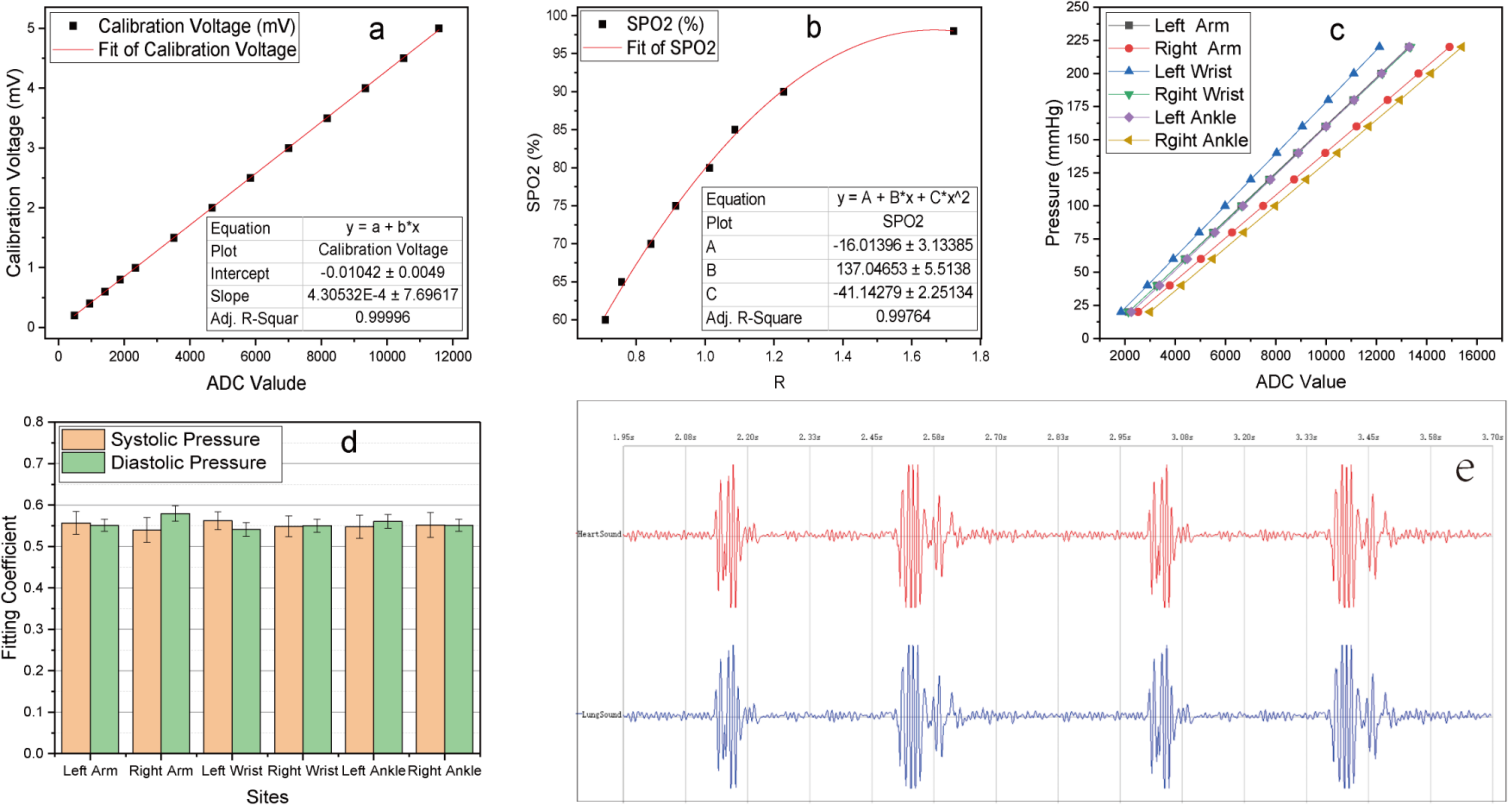
Calibration of key parameters. (a) for ECG amplitude; (b) for blood oxygen saturation; (c) for pressure signals of six cuffs; (d) for fitting coefficient of systolic and diastolic pressure; (e) the waveform of HLSs when simulated normal heart sound is input.

In order to clarify and improve the accuracy of signal measurement in this system, three professional calibration instruments, including SKX-1000 + integrated simulator of ECG and blood oxygen saturation, H2-5000 series blood pressure simulator, and XM-TZY-1 Cardiopulmonary sound simulator, were applied. Among them, the simulator SKX-1000+, made by Xuzhou Mingsheng Electronic Technology Co., LTD in China, provides higher than 2% accuracy in ECG amplitude, frequency, and blood oxygen saturation. As shown in Fig. 3(a), 13 amplitude calibration points of simulated ECG were selected as inputs. Each amplitude calibration point was tested more than 100 cycles, and the average of the ADCV about ECG amplitude for 100 consecutive cycles was fitted to the input ECG amplitude, and the maximum relative standard deviation of 100 cycles was less than 3.2%. By linear fitting, the relationship between the ECG voltage and the ADCV has been obtained, shown the following formula (1).

1 $${{\text{V}}_{{\text{ECG}}}}= - 0.0{\text{1}}0{\text{42}}\,+\,{\text{4}}.{\text{3}}0{\text{532}} \times {\text{1}}{0^{ - \,{\text{4}}}}{\text{ADC}}{{\text{V}}_{{\text{ECG}}}}\left( {{\text{mV}}} \right)$$

Additionally, the maximum relative difference between the voltage calculated by formula (1) and the actual input voltage of ECG was less than 1.5%. Meanwhile, the heart rate was calibrated from 40 bpm to 150 bpm at intervals of 10 bpm, and a maximal difference of 0.12 bpm at 140 bpm was achieved.

The simulator SKX-1000+, providing eight calibration points (60%,65%,70%,75%,80%,85%,90%,98%) about the saturation of peripheral oxygen (SPO2), was also applied to acquire the calculation formula of SPO2 in this system. Each calibration point was measured more than 30 cycles, and the ADCV about BODWSs for 30 consecutive cycles was fitted to the input calibration point. The ratios of the pulsation value to the mean value for each cycle about BODWSs were calculated, and the R value for each period was obtained by dividing the ratio of 660 nm and of 940 nm. The average of the R Values during 30 consecutive cycles was fitted to the input of SPO2, and the maximum relative standard deviation of the 30 cycles was less than 1.9%. By parabola fitting, the relationship between the SPO2 Value and R values was gained, displayed in Fig. 3(b), and expressed by the following formula (2).

2 $${\text{SPO2}}= - {\text{16}}0.0{\text{1396}}\,+\,{\text{137}}0.0{\text{4653R}}\,+\,{\text{41}}.{\text{14279}}{{\text{R}}^{\text{2}}}\left( \% \right)$$

The H2-5000 series blood pressure simulator, made by Xuzhou Mingsheng Electronic Technology Co., LTD in China, provides a ± 0.5mmHg pressure accuracy class, and a 0.1mmHg pressure resolution. During pressure calibration, in order to improve the accuracy and consistency of the pressure measurement at the 6 sites, the gas paths at the 6 sites were connected with an air band, and the gas band pressure was reduced from about 230 mmHg to 20 mmHg by a very slow rate of vent (about 0.1 mmHg/s) at each pressure calibration point. From 220 mmHg to 20 mmHg, each interval of 20 mmHg was a calibration point, so analog-digital conversion values of 6-channel pressure signals at 11 pressure calibration points can be obtained during each measurement, as displayed in Fig. 3(c). In order to improve the accuracy of the pressure measurement, each pressure calibration point was performed 5 times. The 5-times average of ADCV about each-channel pressure signal at every calibration point was fitted to the input pressure, and the maximum relative standard deviation of 5-times ADCV among the 6-channel pressure signals was less than 0.4%, which means that the pressure measurement of this system has good stability. By linear fitting, some parameters between pressure and ADCV about each pressure signal, such as intercept value, slope value, and adjusted R-square, were obtained. These fitting parameters suggested that there is a good linear relationship between the ADCV and the pressure value (see Supplementary Table S2 for the details).

The maximum relative difference between the pressure value calculated by the formula (2) and the actual input value was less than 0.16% except for 20mmHg with about 1.7%.

Furthermore, the H2-5000 simulator also has the function of blood pressure simulation, and can provide seven kinds of standard blood pressure simulation, and each kind of the pressure simulation was also performed 5 times. By normalized oscillographic method, the fitting coefficients of systolic and diastolic pressure about the six sites were acquired. The averages and standard deviations are illustrated in Fig. 3(d) (see Supplementary Table S3 for the details).

XM-TZY-1 Cardiopulmonary sound simulator is made by Shanghai Xinman Science Teaching Equipment Co., LTD in China, and can generate 34 typical heart and breath sounds. Here, only the normal heart sound was used to verify the consistency of the heart and lung sound signal channels in this system. Because the amplitude of heart and lung sounds is greatly affected by the weight of the human individual, and their amplitudes are of no practical significance in the analysis.

The simulated normal heart sound generated by the simulator was input to the heart sound and lung sound sensor at the same time, and the output waveform of the two-channel signals was acquired and displayed in Fig. 3(e), which indicates that the signal pathways of heart sound and lung sound have good consistency.

In general, through the above calibration and testing, the system can obtain high precision and good stability for the measurement of each channel signal.

### Signal collection

Before signal collection, their basic information is acquired and input into the computer-application software of the system. The basic information covers name, gender, age, height, weight, occupation, hypertension or not, diabetes or not, distances between wrist and arm as well as wrist and middle fingertip, distance between ankle and middle tiptoe, blood pressure and heart rate measured using a electronic sphygmomanometer OMRON HEM-8102. When measuring, the subject is instructed to lie on the bed, and all sensors are placed at designated locations illustrated in Fig. 1(a). The entire signal collection, which lasts about 10 minutes, can be completed by running the computer-application software of this system on a notebook/desktop computer connected to the signal collector via a USB cable connection. Additionally, to enhance stability and security, and to minimize power interference, a rechargeable lithium battery with a capacity of 7.4 V & 15.0 Ah is employed for power supply.

**Fig. 4:**
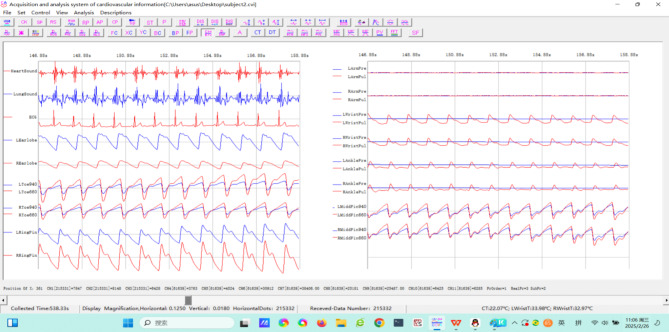
The waveform of 27-channel signals from moment t = 146.88 s to t = 156.88 s about subject2.

In order to assess the reliability and practicality of our system, sixteen volunteers (see Supplementary Table S4 for their basic information) were recruited. Meanwhile, informed consent was obtained from all subjects. Additionally, all experimental protocols were approved by the Ethics Committee of Chongqing Medical University. And we state that all experimental methods were performed in accordance with the relevant guidelines and regulations. For intuitively exhibiting the signal acquisition results, the subject2 is presented as an example, Fig. 4 displays an excerpted waveform spanning from moment t = 146.88 s to t = 158.88 s within this overall recording duration (see Supplementary Table S5 for the abbreviation and their full names in Fig. 4, and Fig. S2 the complete waveform of all 27-channel signals during the whole collection process).

### Data analysis method

Many studies have been conducted to analyze signals related to ECG, HS, LS, pressure pulse, PPG, and BODWS. In this study, out of 27 channels all can be used for time-domain and frequency-domain analysis except for the 6 pressure signals. the 21-channel signals, including six-channel pressure pulse, four-channel BODWSs (eight-channel signals), four-channel PPGSs, one-channel ECG, one-channel HS, and one-channel LS.

#### Time-domain analysis

In time domain, Firstly, for minimizing interference, a 5-point smoothing filter is applied to process all raw signals. Secondly, the R-peak positions of ECG are obtained by a special method. Subsequently, each interval time between two adjacent R-peak positions during each RMPS can be computed, and the interval time that meets the special conditions serves as a calculated cycle. Then all 21-channel signals can be analyzed based on the calculated cycles. In this system, the analysis, described as follows, is mainly to extract various parameters, which should be able to reflect the waveform characteristics and its change characteristics of each signal as far as possible in order to effectively realize the diagnosis and health assessment of CVDs.

1. Obtain the calculated cycles.

After smoothing for the raw ECG signal, each R-peak position of ECG signal can be obtained by the classical differential threshold method^51^. Then, the time between two adjacent R-peaks of the ECG signal during all RMPSs can be calculated according to the sampling frequency, and sort the time from smallest to largest. Furthermore, the average value of its middle 50% time interval is taken as the base time, and only the time between two adjacent R-peaks, which is 0.7 to 1.6 times the base time, is regarded as the calculated cycle (see Supplementary Method S1 for the details).

(2) Compute characteristic parameters of HS signal.

Based on above obtained each calculated cycle (ECC), calculate the amplitude of the first and second heart sounds of ECC about HS signal, and their ratio, and the normalized maximum amplitude-change rate (NMACR, the ratio of maximum amplitude-change rate and maximum value) of ECC about HS signal, the ratio of the mean and maximum value during each one third time segment of ECC about HS signal, and their average, standard deviation during the RMPSs, etc. .

(3) Calculate characteristic parameters of LS signal.

Based on the ECC, withdraw the maximum value, NMACR, the ratio of the mean and maximum value during each one third time segment of ECC about lung sound signal, and their average, standard deviation during the RMPSs, and so on.

(4) Withdraw characteristic parameters of ECG.

**Fig. 5:**
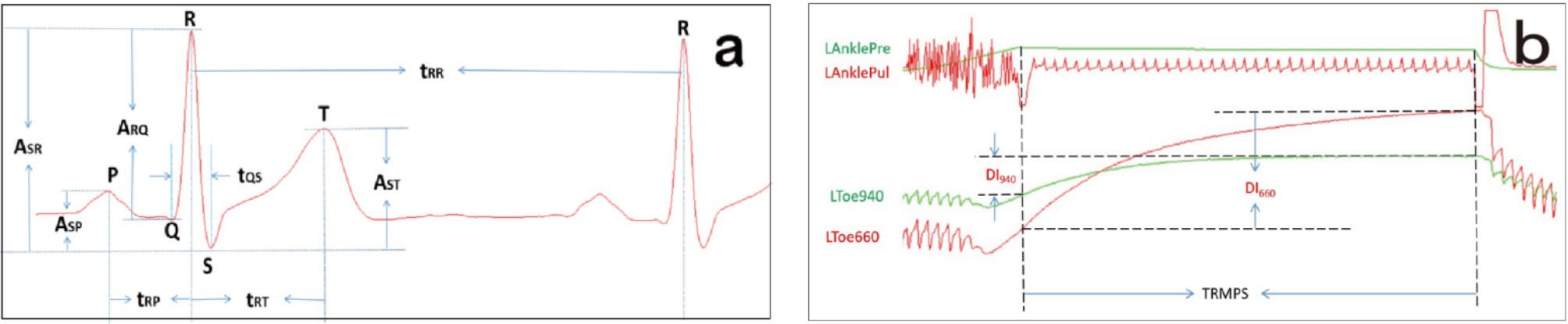
Schematic diagram of some characteristic parameters. (**a**) about ECG; (**b**) about BODWSs.

Based on ECC, calculate ECG parameters, shown in Fig. 5(a), such as the time between adjacent R-peaks of ECG (t_RR_), the amplitude difference (A_SR_) between S-wave and R-wave of ECG, and A_RQ_, A_SR_, A_ST_, the ratio of A_RQ_ and A_SR_, and the ratio of A_SP_ and A_ST_, the time t_QS_ between Q-wave and S-wave of ECG, and t_RP_, t_RT_, the ratio of t_RP_ and t_RT_, NMACR of QR-wave and SR-wave, as well as their average, standard deviation during the RMPSs, and son on.

(5) Obtain respective parameters of 18-channel signals.

Based on the ECC, compute the following parameters for the remaining 18-channel signals (excluding ECG, HS, and LS from the 21 channels): the difference (A_d_) between maximum and minimum value, NMACR, K Value (the ratio of mean to difference between the amplitude maximum and minimum values), the time from R-peak of ECG to positions of maximum value, minimum value, and maximum-change rate during the ECC, and the average, standard deviation, normalized standard deviation (NSD, the ratio of standard deviation and its average) of each parameter during special RMPSs, some other ratio and their normalized standard deviations (NSDs) during the some RMPSs, etc. (see Supplementary Method S2 for the details of the respective parameters).

(6) Calculate other type parameters.

Based on each channel computed parameters, calculate the ratios of the same parameter of left-and-right signals, their standard deviations, and other parameters, such as normalized average-change rates of four-channel BODWSs during RMPS4 and RMPS7 by formula (3), ROS by formula (4), NROS by formula (5), pulse wave velocity (PWV) by formula (6), and so on.

3 $$\:{\text{A}\text{C}\text{R}}_{{\uplambda\:}}=\frac{{\text{A}}_{{\uplambda\:}}}{{{\text{D}\text{I}}_{{\uplambda\:}}\text{T}}_{\text{R}\text{M}\text{P}\text{S}}}$$

4 $$\:\text{R}\text{O}\text{S}=\frac{{\text{A}\text{C}\text{R}}_{660}}{{\text{A}\text{C}\text{R}}_{940}}$$

5 $$\:\text{N}\text{R}\text{O}\text{S}=\frac{{\text{A}\text{C}\text{R}}_{940}\times\:{\text{M}\text{D}1}_{660}}{{\text{A}\text{C}\text{R}}_{660}\times\:{\text{M}\text{D}1}_{940}}$$

6 $${\text{PWV}} = {\text{L}}_{{{\text{ab}}}} {\text{/t}}_{{{\text{ab}}}}$$

As shown in Fig. 5(b), Where λ presents wavelength 940 nm–660 nm, DI_λ_ expresses differences between the last and the first data of BODWSs about 940 nm–660 nm during RMPS4 or RMPS7, T_RMPS_ is the continuance time of RMPS4 or RMPS7. ACR_λ_ presents the average-change rates of 940 nm–660 nm among BODWSs. A_λ_ is the average change at the corresponding wavelength during RMPS4 or RMPS7, ROS is the ratio of ACR_660_ to ACR_940_, NROS expresses the normalized ROS, MD1_660_ and MD1_940_ respectively present the means of the maximum-and-minimum difference of corresponding wavelength in each cardiac cycle during RMPS1, L_ab_ equals to the length of site a and b, t_ab_ is the mean of the time differences between R-wave to maximum amplitude-change rate (RWMACR) about calculated signals at site a and b during ACCs. For fingers and toes, the optoelectronic signal (OS) of 640 nm is used, and for wrists, arms, and ankles, the PPS is applied. The L_heart_to_ankle_=0.8129×H + 12.328 cm, L_heart_to_arm_=0.2195×H-2.0734 cm^52^, and H represents the subject’s height, and other distances are measured manually.

Additionally, according to all calculated cycles during RMPS2, and RMPS4, calculate systolic and diastolic blood pressure of two-side arms, wrists, and ankles by the oscillometry method^53,54^, and their average blood pressure. In addition, withdraw the other parameters of each channel signal during the entire duration of RMPS2 and RMPS4, such as the maximum-change rate during the entire calculated RMPS, and so on.

#### Frequency-domain analysis

In the system, the fast Fourier transform (FFT) is employed to obtain the frequency distribution of the 21-channel signals during the seven RMPSs. Then, Calculate the heart rate (HR) of each RMPS according to the ECC, and subsequently, the positions from 1 to 10 times heart rate (THR) along with their corresponding selected frequency ranges were graphically represented (see Supplementary Material S1 for details). Based on the following formula (7) to formula (10), compute AveF_ijn_, MaxF_ijn_, RAMF_ijn_, and RMMF_ijk_ for the 21-channel signals during all RMPSs.

7 $$\:{\text{A}\text{v}\text{e}\text{F}}_{\text{i}\text{j}\text{n}}=\text{E}\left({\text{F}\text{F}\text{T}\text{D}}_{\text{i}\text{j}}\right(\text{m}\left)\right|\text{m}\in\:{\text{F}\text{R}}_{\text{i}\text{j}\text{n}})$$

8 $$\:{\text{M}\text{a}\text{x}\text{F}}_{\text{i}\text{j}\text{n}}=\text{M}\text{a}\text{x}\left({\text{F}\text{F}\text{T}\text{D}}_{\text{i}\text{j}}\right(\text{m}\left)\right|\text{m}\in\:{\text{F}\text{R}}_{\text{i}\text{j}\text{n}})$$

9 $$\:{\text{R}\text{A}\text{M}\text{F}}_{\text{i}\text{j}\text{n}}=\frac{{\text{A}\text{v}\text{e}\text{F}}_{\text{i}\text{j}\text{n}}}{{\text{M}\text{a}\text{x}\text{F}}_{\text{i}\text{j}\text{n}}}$$

10 $$\:{\text{R}\text{M}\text{M}\text{F}}_{\text{i}\text{j}\text{k}}=\frac{{\text{M}\text{a}\text{x}\text{F}}_{\text{i}\text{j}\text{k}}}{{\text{M}\text{a}\text{x}\text{F}}_{\text{i}\text{j}1}}$$

Where $\:\text{E}$() and Max() respectively express averaging and maximizing operations, FFTD_ij_(m) presents the data sequence of signal i during RMPS j after performing FFT, m is the sequence number among data sequences, i expresses the sequence number of a channel signal among above 21 channels, ranging from 1 to 21, j is the sequence number of an RMPS among the seven RMPSs, n expresses the serial of THR with the value ranging from 1 to 10, and k also presents the times of THR, ranging from 2 to 10 (see Supplementary Material S1 for details).

If the number of FFT data about signal i during RMPS j is N_ij_, and f_s_ is the sampling frequency of signal i, record f_s_/N_ij_ as FN_ij_, $\:{\text{F}\text{R}}_{\text{i}\text{j}\text{n}}$ falls within a range defined by Round((*n*−0.2)×FN_ij_ ×HR) to Round ((*n* + 0.2)×FN_ij_]×HR), and the Round () denotes an integer rounding operation.

## Results and discussion

Through the analysis of 27-channel signals during seven RMPSs, more than 1000 CVPs, encompassing both established clinical significance and novel indices, can be derived. Due to space limitations, only some meaningful findings are presented in this paper.

### Exemplary results of a subject

In order to demonstrate the system’s performance, some exemplary analysis results of the subject2, a 24-year-old male subject with a height of 171 cm and weight of 65.4 kg (see Supplementary Table S4(b) for his more basic information), are presented below.

#### ECG

**Fig. 6:**
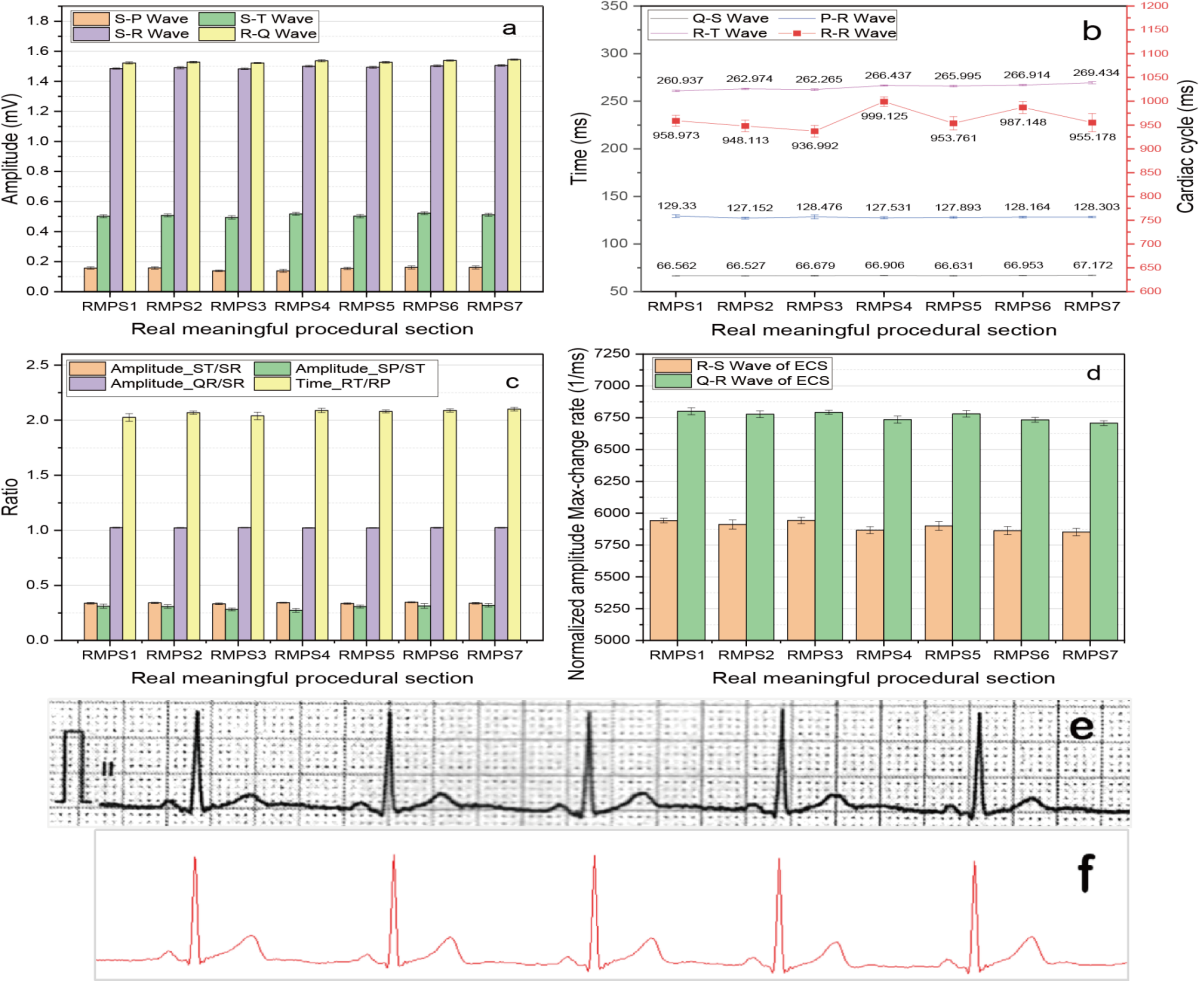
Some analysis results of ECG about the subject2. (**a**) The amplitude differences between S-wave and P-wave, S-wave and T-wave, S-wave and R-wave, R-wave and Q-wave of ECG; (**b**) The time between Q-wave and S-wave, P-wave and R-wave, R-wave and T-wave, R-wave and R-wave of ECG; (**c**) The amplitude ratios of ST/SR, SP/ST, QR/SR, RT/RP, and the time ratio of RT/RP; (**d**) Normalized amplitude max-change rate of R-wave to S-wave, and Q-wave to R-wave of ECG; (**e**) ECG of lead II obtained by electrocardiograph; (**f**) ECG of lead II obtained by the system.

Many valuable cardiovascular parameters can be derived from ECG, such as heart rate, cardiac cycle, time between Q-wave and S-wave, the amplitude differences between S-wave and R-wave, between S-wave and Q-wave, and so on. In Fig. 6, some analysis results of ECG about the subject are shown.

These fourteen characteristic parameters and their standard deviations of ECG, exhibited in Fig. 6(a)-(d), have minimal changes from RMPS1 to RMPS7, which indicates that pressure applied on both arms, wrists, and ankles has negligible impact on ECG measurements for this subject, and the system has good stability for ECG measurement. In addition, Fig. 6(e) and Fig. 6(f) respectively show the waveform of lead II of ECG obtained by the electrocardiograph in the physical examination center of the First Affiliated Hospital of Chongqing Medical University and by this system, the appearances of waveform are very similar, which further indicates that the accuracy and reliability of ECG acquisition about this research system are good.

#### LHS

**Fig. 7:**
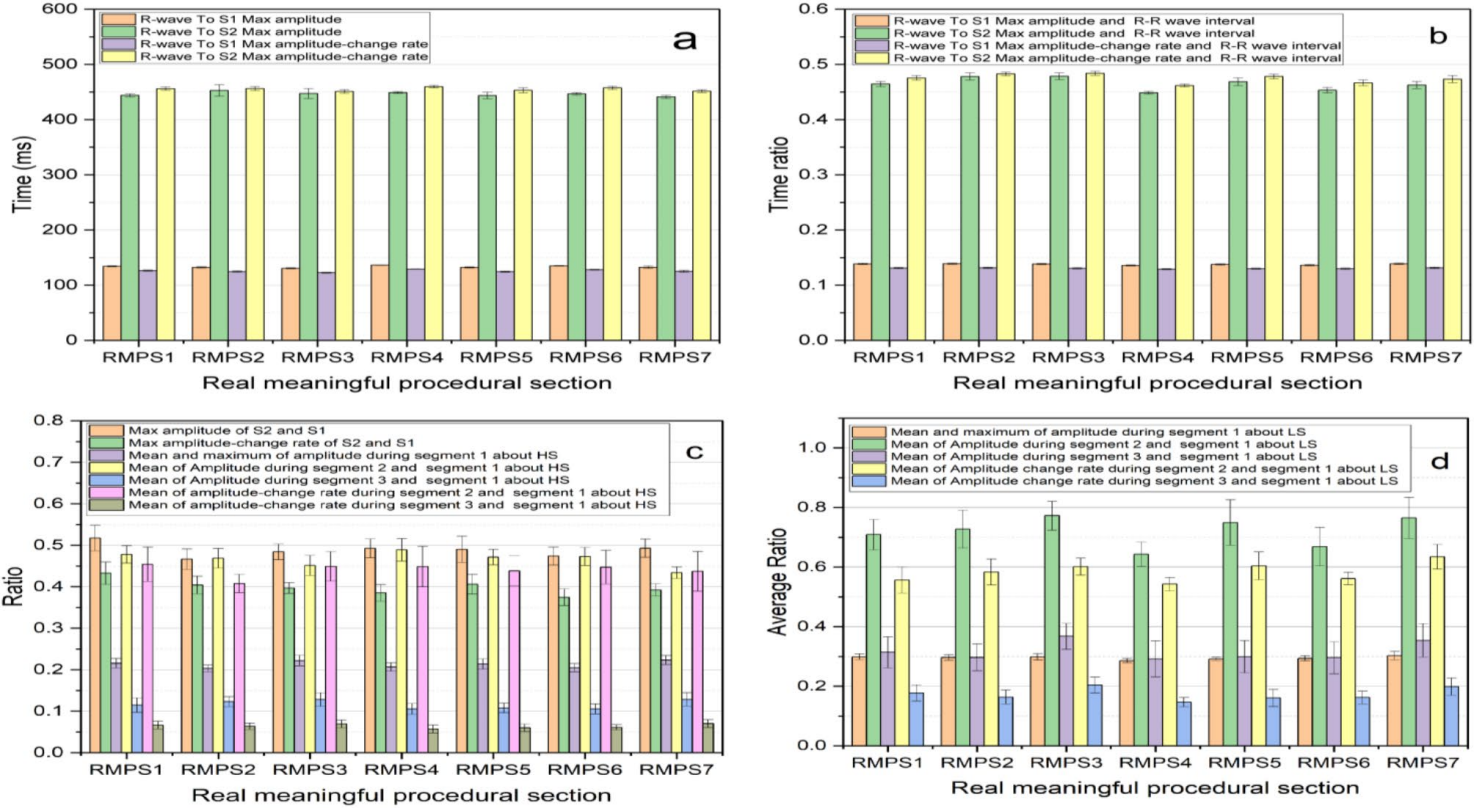
Some characteristic parameters of HS and LS about the subject. (**a**) The time between R-peak and maximum amplitude of S1, R-peak and maximum amplitude of S2, R-peak and maximum amplitude-change rate of S1, R-peak and maximum amplitude-change rate of S2; (**b**) The ratios between the time shown in Fig. 7(a) and R-R wave interval; (**c**) The ratios of maximum amplitude and maximum amplitude-change rate of S2 and S1, and the mean and maximum of amplitude during segment 1, of the amplitude mean during from segment 2 to segment 3 and segment 1, the ratios of mean and maximum of amplitude-change rate during from segment 2 to 3 and segment 1 about HS; (**d**) The ratios of the mean and maximum of amplitude during segment1, of the amplitude mean during from segment 2 to segment 3 and segment 1, the ratios of mean and maximum of amplitude-change rate during from segment 2 to 3 and segment 1 about LS.

The system enables the extraction of various characteristic parameters of lung and heart sound (LHS), such as the means and standard deviations of maximum value and NMACR during the seven RMPSs. Figure 7 illustrates a subset of these parameters.

The eight parameters, depicted in Fig. 7(a) and Fig. 7(b), exhibit minimal variations, and the remaining 12 parameters displayed in Fig. 7(c) and Fig. 7(d) demonstrate relatively insignificant changes across the seven RMPSs investigated. Additionally, some other parameters about HS and LS (such as the ratios of mean and maximum amplitude-change rate from segments 1 to 3) also indicate insignificant changes (see Supplementary Fig. S3 for other parameters). All these findings suggest that pressure applied on both arms, wrists, and ankles has no discernible impact on heart and lung sound signals for this subject. And they also indicates that the system has good stability for LHS signals.

#### Photoplethysmographic-and-pressure pulse signals

**Fig. 8:**
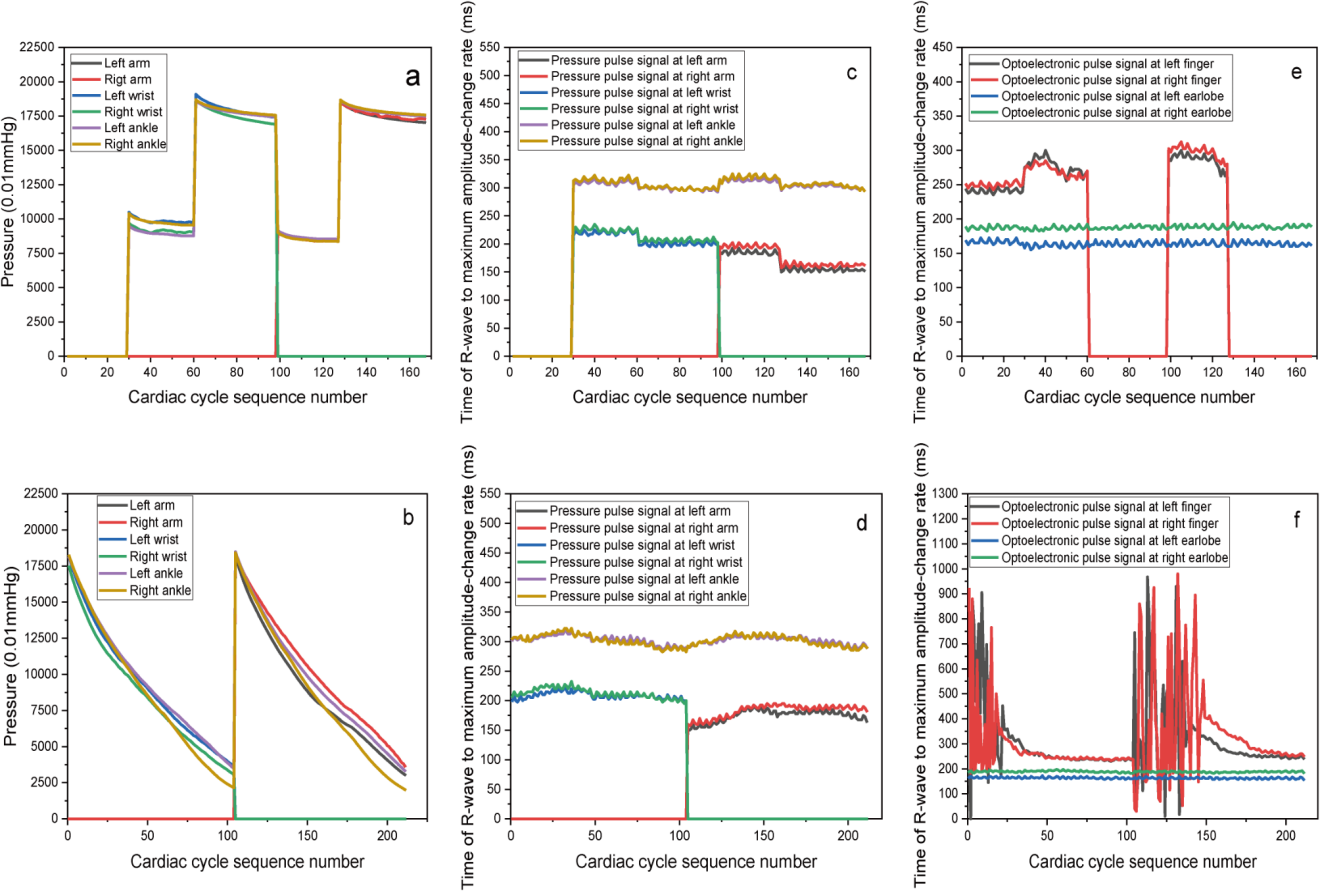
Some characteristic-parameter distribution of six-channel PPS and four-channel optoelectronic pulse about the subject. (**a**) The pressure distribution of six-channel pressure signals during RMPS1, RMPS3-RMPS4, RMPS6-RMPS7; (**b**) The pressure distribution of six-channel pressure signals during RMPS2 and RMPS5; (**c**) The time of R-peak and maximum amplitude-change rate of six-channel pressure pulse signals during RMPS1,RMPS3-RMPS4,RMPS6-RMPS7;(**d**) The time of R-peak and maximum amplitude-change rate of six-channel pressure pulse signals during RMPS2 and RMPS5; (**e**) The time distribution of RWMACR about optoelectronic pulse signals at both-side fingers and earlobes during RMPS1, RMPS3-RMPS4, RMPS6-RMPS7; (**f**) The time distribution of RWMACR about optoelectronic pulse signals at both-side fingers and earlobes during RMPS2 and RMPS5;

**Fig. 9:**
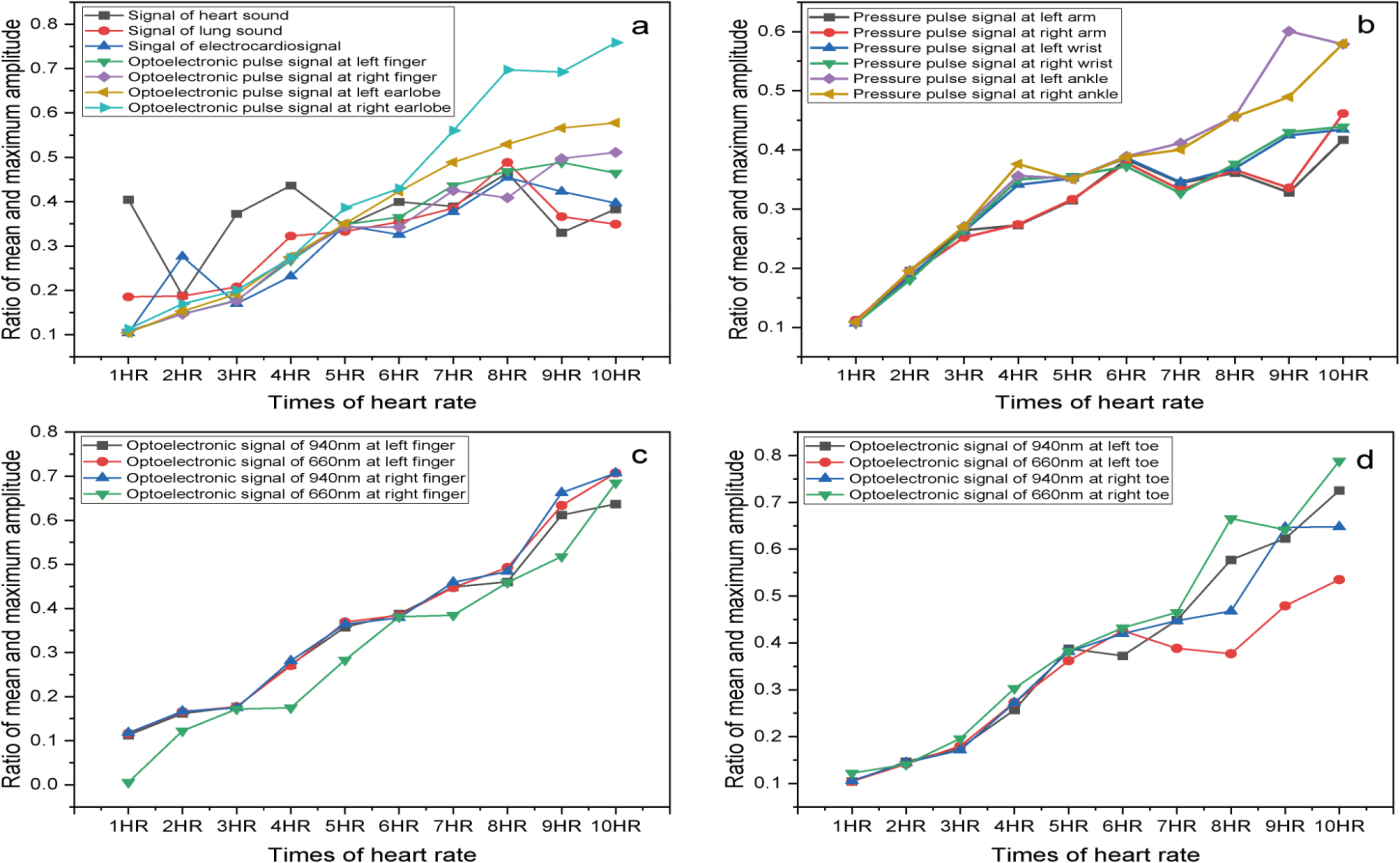
The ratios of mean and maximum amplitude in the frequency domain of collected 21-channel signals about the subject. (**a**) The ratios about HS, LS, ECG, and optoelectronic pulse signals at both-side fingers and earlobes during RMPS1; (**b**)The ratios about pressure pulse signals at both-side wrists and ankles during RMPS3, at both-side arms during RMPS6; (**c**) The ratios about optoelectronic pulse signals at both-side fingers during RMPS1; (**d**)The ratios about optoelectronic pulse signals at both-side toes during RMPS1.

Based on the photoplethysmographic-and-pressure pulse signals, various cardiovascular parameters can be obtained during ECC, including the mean values, the time of R-peak to maximum value, and of RWMACR about six-channel pressure and four-channel optoelectronic pulse signals, and others. Some of these parameters are illustrated in Figs. 8 and 9. Table 3 presents the associations among RMPSs, the serial number of cardiac cycle sequence, and the figure.

**Table 3:** The relationships among RMPSs, serial number of cardiac cycle sequence, and figure.

Serial number	RMPS1	RMPS3	RMPS4	RMPS6	RMPS7	RMPS2	RMPS5
Cardiac cycle sequence	1–28	29–59	60–97	98–126	127–166	1−104	105–211
Figure	Figure 8(a), Fig. 8(c), Fig. 8(e);					Figure 8(b), Fig. 8(d), Fig. 8(f)	

The mean-pressure changes of ECC, displayed in Fig. 8(a) and Fig. 8(b), are less than ideal. It would be ideal if the four RMPSs (RMPS3, RMPS4, RMPS6, and RMPS7) maintain a constant pressure, while RMPS2 and RMPS5 keep a linear, slow venting process. And these require further improvement in the future. According to the findings depicted in Fig. 8(c) and Fig. 8(d), variations in RWMACR time have a minor impact on wrist, arm, and ankle pressures. Figure 8(e) and Fig. 8(f) indicate that the RWMACR time of optoelectronic signals (OSs) at both earlobes remains unaffected by pressure applied to the arms, wrists, and ankles on both sides, which demonstrates that this value is a relatively stable value for subject2. However, smaller effects are observed when pressures remain below approximately 100 mmHg at wrists and 90 mmHg at both arms and ankles. Overall, as illustrated in Fig. 8, it can be inferred that the time of the 10-channel signals may serve as a relatively stable individual parameter.

#### Frequency domain

**Fig. 10:**
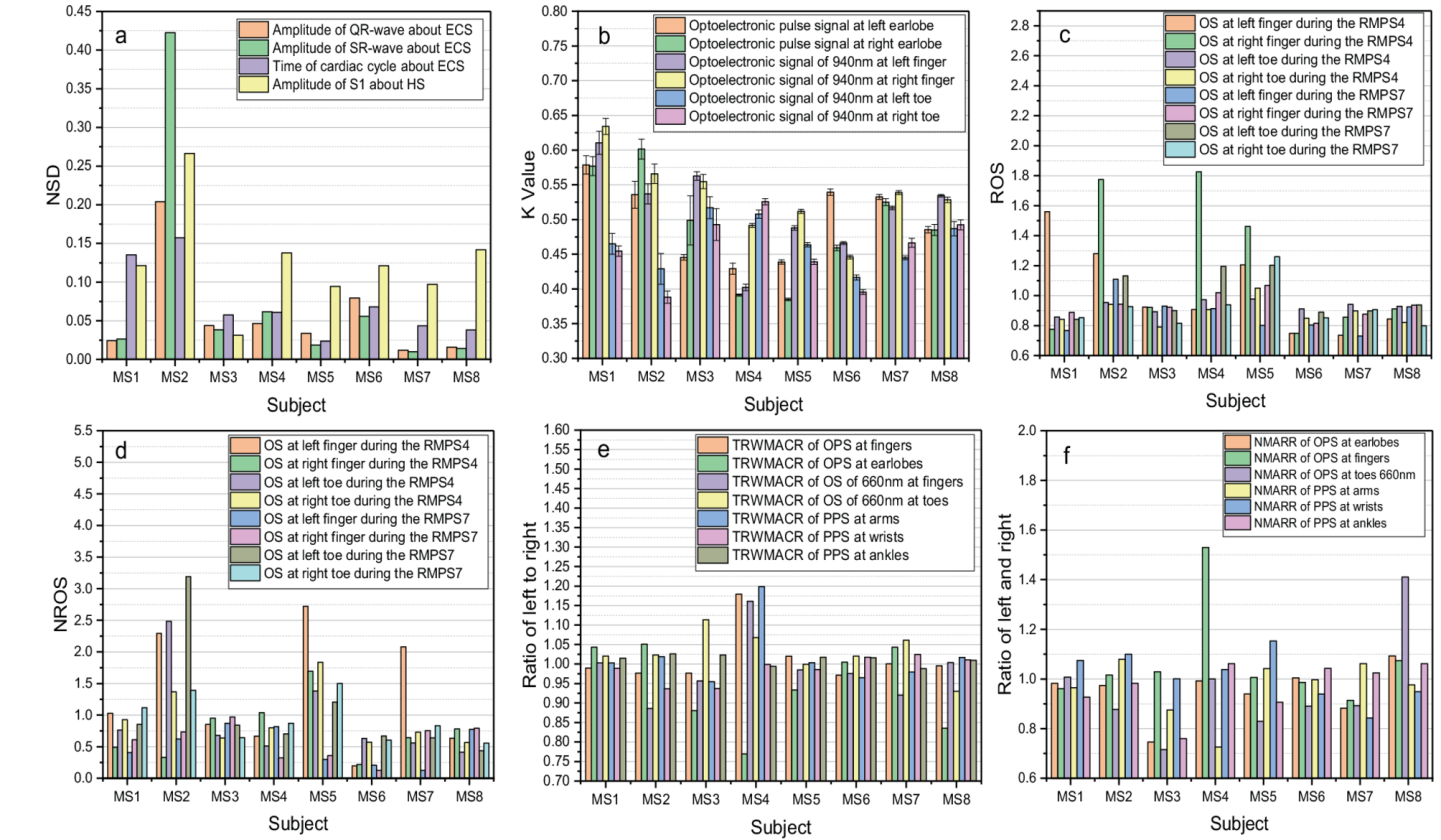
Some parameters about the eight subjects. (**a**) NSD of the amplitude of QR-wave and SR-wave about ECG, the time of the cardiac cycle about ECG, and amplitude of S1 about HS during from RMPS1 to RMPS7; (**b**) the K value of OPSs at left-and-right earlobes, and of OSs at both-side fingers during the RMPS1; (**c**) the ROS of OSs at fingers and toes during RMPS4 and RMPS7; (**d**) the NROS of OSs at fingers and toes during RMPS4 and RMPS7; (**e**) the ratios of left to right about TEWMACR of OPSs at fingers and earlobes during RMPS1, of OSs of 660 nm at fingers and toes during RMPS1, and of PPSs at wrists during RMPS3, and of PPSs at arms, and ankles during RMPS6; (f) the ratios of left to right about NMARR of OPSs at fingers and earlobes during RMPS1, of OSs of 940 nm at toes during RMPS1, and of PPSs at wrists during RMPS3, and of PPSs at arms, and ankles during RMPS6.

The system is capable of providing frequency domain analysis results for each subject, encompassing the maximum amplitude in one-times HR, the ratios of mean and maximum amplitudes within a specified frequency range, and the normalized maximum amplitude ranging from 2 HR to 10 HR. Figure 10 displays the ratios of mean and maximum amplitudes in the frequency domain for the 21-channel signals, computed using formula (9) during RMSP.

According to Fig. 9, there is a similar trend observed in the ratios of optoelectronic pulse signals (OPSs) of the earlobes and fingers, pressure pulse signals (PPSs) at arms, wrists, and ankles on both sides, as well as OSs at fingers and toes with wavelengths of 660 nm and 940 nm. Notably, significant differences can be observed in Fig. 9(a), which represents OPSs of left and right earlobes, and these align with the differences observed in the time domain shown in Fig. 4. This suggests that these ratios may offer a quantitative description of disparities between similar left-right signals.

In addition, for testing the stability of our designed system, the subject2 was measured five times. The results, such as the average time of RWMACR, NMACR, and K value of the 18-channel signals, blood pressure, and PWV (see Supplementary Material S2 for the details of five-times tested results about the subject2 ), exhibit a good consistency, which means that this system have a certain level of reliability.

### Exemplary results of multiple subjects

As mentioned previously, it is evident that the system can provide comprehensive information in both time and frequency domains for each subject. To further validate its significance, sixteen subjects were measured. From among them, eight subjects (hereinafter referred to as MS1 to MS8, see Supplementary Table S4(a) for their basic information and Fig. S4 for their waveform of 27-channel signals) were selected, and Some exemplary results are exhibited in Fig. 10.

In Fig. 10(a), the NSD, the ratio of standard deviation and its average during the time from RMPS1 to RMPS7, reflects the relative dispersion of the data. Among the eight subjects, MS1 and MS2 exhibit larger normalized standard deviations (NSDs) in their cardiac cycles compared to others, which indicates greater changes in their cardiac cycles. These observations are consistent with the arrhythmia (see Supplementary Table S4(a) for their basic information and Fig. S4 for their waveform of 27-channel signals). Therefore, based on this parameter, arrhythmia can be assessed for each subject. As shown in Fig. 10(a), NSDs of the other three parameters about the subject MS2, which reflect the amplitude relative variations of QR-wave and SR-wave about ECG, and of S1 about HS among different cardiac cycles, are much larger than that of other subjects, and these are consistent with that the subject MS2 is premature beat patients. Figure 10(b) illustrates the distribution of K values about the six-channel signals during RMPS1. These K values roughly reflect signal waveform shape characteristics. Generally, a rounder top of a signal waveform corresponds to a higher k value. Based on Fig. 10(b), good symmetry is observed between left-and-right corresponding signals at earlobes, fingers, and toes for subjects MS1, MS7, and MS8 about their K values. However, the K values at earlobes for other subjects show significant differences between both sides which may be associated with carotid stenosis or increased right vertebral artery flow resistance. Particularly, the subject MS3 has an approximate right internal carotid artery stenosis rate of 50–70%. These findings suggest that OPSs obtained from earlobes maybe can provide valuable information, like carotid artery stenosis and cardiovascular evaluation. The ROSs of MS3, RMS6-RM8 exhibit minimal changes as depicted in Fig. 10(c), indicating that the alteration rates of OSs at 660 nm and 940 nm wavelengths are approximately equal between fingers and toes when blood flows are impeded. However, noticeable changes are observed for other parameters. Notably, there is a substantial disparity in the effect of pressure on SORs between fingers at the wrists and arms. The NROSs of MS2, MS5, and MS7, displayed in Fig. 10(d), are different. The ratios of left to right TRWMACR shown in Fig. 10(e) provide an approximate reflection of bilateral symmetry. Among the participants, significant differences in TRWMACR are observed between the left and right sides for MS4 at fingers, earlobes, and arms. In Fig. 10(f), the ratios of left to right NMARR (normalized maximum-amplitude rise rate) are equal to dividing maximum-amplitude rise rate by the difference between maximum and minimum during ECC. Obvious differences exist between both sides for SM3 at earlobes, toes, and ankles, for MS4 at fingers and arms, as well as for MS8 at toes regions. The analysis, presented in Fig. 10, demonstrates considerable inter-subject variability across various parameters with some having known clinical significance such as NSD while others require further investigation through extensive clinical trials.

**Fig. 11:**
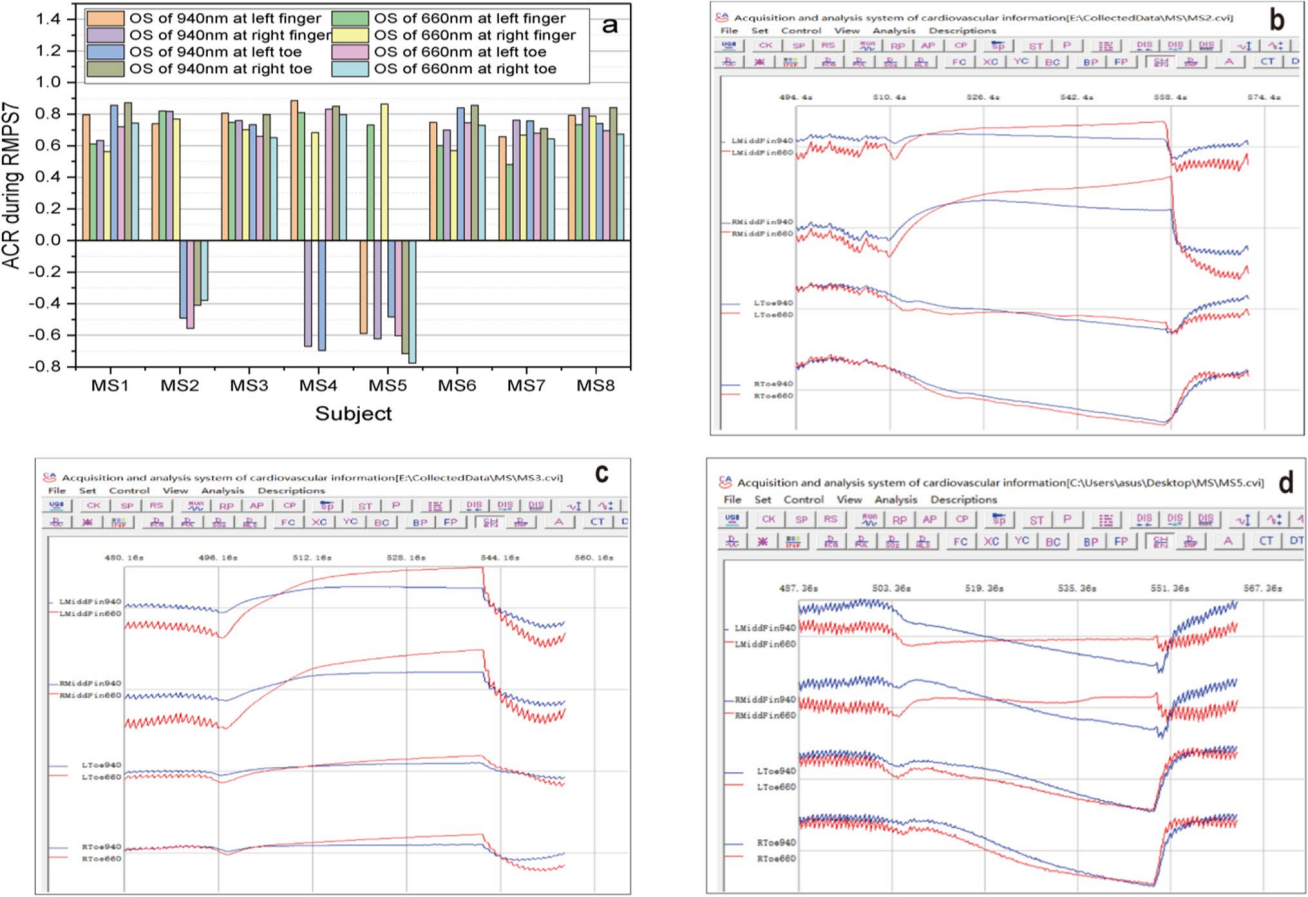
ACR parameter about the eight subjects and waveform of some subjects during RMPS7. (**a**) ACR parameter about the eight subjects; (**b**) waveform of BODWSs about MS2; (**c**) waveform of BODWSs about MS3; (**d**) waveform of BODWSs about MS3.

As displayed in Fig. 11, some BODWSs about the eight subjects show an obvious opposite variation trend during RMPS7 (the stimulation of blocking blood pressure). For example, in Fig. 11(a), some ACR (calculated by formula (3)) values of BODWSs about MS2, MS4, and MS5 are different from that of other subjects. To intuitively show the specific variation difference of the signal, the specific waveform diagrams of MS2, MS3, and MS5 during the process are presented in Fig. 11(b) to Fig. 11(d). The variation trends of BODWSs about MS3 are normal. While BODWSs at both side toes about MS2 and at both side fingers and toes about MS5 show obvious abnormal changing trend. And the symmetry of change trends about the left and right of MS2 and MS5 was different, while the trend of MS3, which is normal, displays a better symmetry at the left and right. Therefore, the stimulation of blocking blood pressure provides a new method for studying metabolic conditions of fingers and toes, as well as CVDs. Meanwhile, it indicates that information that cannot be obtained by traditional methods can be obtained through pressure stimulation.

**Table 4:** Comparison of acquisition methods and their results of cardiovascular information.

Stimulus mode	Number of signal channels and types	The types and sites of collected signals	Results (accuracy, noise, novelty)	Ref. Year
No Stimulus	7 channels 2 types	ECG (one-channel, Not stated site) ; PPG at both earlobes, fingers , and toes;	Accuracy: not stated; Noise: a good stability and reliability; Novelty: 1. Synchronously gathering 7-channel physiological signals in a non-invasive way. 2. Provide a promising tool for diagnosis in CVDs.	^34^ 2006
No Stimulus	2 channels 2 types	ECG at lead II (3-lead ECG); Heart sound	Accuracy: The system noise in the ECG channel was less than 30 µV and the frequency response ranged from 0.05 to 100 Hz, while the signal-to-noise ratio in the system’s heart sound channel was 25.6 dB. Noise: a good stability and reliability; Novelty: 1. Synchronously gathering 2-channel physiological signals in a non-invasive way. 2. Helping to achieve screening of CVDs.	^35^ 2024
No Stimulus	4 channels 3 types	ECG at lead II (3-lead ECG); PPG at left index finger; PPW at both-side wrists;	Accuracy: not stated; Noise: a good stability and reliability; Novelty: 1. Synchronously gathering four-channel physiological signals in a non-invasive way. 2. Provide a promising tool for tri-modal diagnosis in CVDs.	^17^ 2018
No Stimulus	3 channels 3 types	ECG at left and right arms, right leg (3-lead ECG); PPG at left middle finger; PPW at left wrist;	Accuracy: The mean absolute errors of systolic and diastolic blood pressure prediction reached 0.90 mmHg and 2.47 mmHg; Noise: a good stability and reliability; Novelty: 1. Synchronously gathering 3-channel physiological signals in a non-invasive way. 2. Proposes a noninvasive blood pressure prediction method for CVDs.	^29^ 2025
Non-pressure; Gradual-decrease pressure; Fixed constant pressure.	24 channels 5 types	ECG at lead II (3-lead ECG); PPG at both middle fingers; BODWSs at both Index fingers; PPW at both wrists, arms, and ankles; HS at pulmonary auscultation area;	Accuracy: not stated; Noise: a good stability and reliability; Novelty: 1. Synchronously gathering 24-channel physiological signals in a non-invasive way. 2. Provide a promising tool for multi-modal diagnosis in CVDs; 3. Pressure stimulus is applied.	^9^ 2017
Non-pressure; Gradual-decrease pressure Personalized maximum-pulse amplitude pressure; Blocking blood flow pressure.	27 channels 6 types	ECG at lead II (3-lead ECG); PPG at both side earlobes and the fourth fingers; BODWSs at both side middle fingers and middle toes; PPW at both side wrists, arms, and ankles; HS at the auscultatory area of the tricuspid valve; LS at anterior superior thoracic right auscultation area.	Accuracy (maximum relative difference compared with verification-and-calibration professional instruments ) : ECG, less than 1.5%; Heart Rate (40–150 bpm), not more than 0.12 bpm; SPO2, less than 0.9%; Pressure, not more than 1.7%; Noise: a good stability and reliability; Novelty: 1. Synchronously gathering 27-channel physiological signals in a non-invasive way. 2. Provide a promising tool for multi-modal diagnosis in CVDs. 3. Personalized pressure stimulus is applied.	This work

PPW (Pressure pulse wave).

In summary, the system is capable of applying differential pressure to six cuffs positioned on the left and right arms, wrists, and ankles while simultaneously capturing 27-channel non-invasive physiological signals. Preliminary test results indicate that the system exhibits excellent stability and reliability. Additionally, as outlined in Table 4, our designed system exhibits notable innovation. By straightforward analysis, the system can obtain both established cardiovascular parameters with known clinical significance and novel parameters with potential clinical implications. Furthermore, through clinical comparative studies, this system is anticipated to generate new clinically valuable evaluation parameters for cardiovascular function assessment, and provide a more comprehensive set of multi-type signal parameters for investigating diagnostic and evaluation models of cardiovascular diseases (CVDs). Notably, the pressure applied by this system is comparable to that of existing electronic blood pressure monitors and arteriosclerosis devices (such as Omron BP-203RPEIII), albeit through a distinct methodology. Additionally, the system has received approval from the Ethics Committee of Chongqing Medical University in China, thereby ensuring its safety.

## Conclusion

In this study, we have proposed a novel 27-channel signal acquisition-and-analysis system incorporating pressure stimulus to maximize the acquisition of CVI. Although only selected results are presented in this paper, they provide sufficient evidence that our developed system can simultaneously obtain a multitude of CVI, including established clinical parameters as well as newly proposed parameters in both time and frequency domains, utilizing our introduced seven RMPSs. However, further research is necessary to fully elucidate the specific clinical value of the newly proposed parameters. Overall, our system offers a promising approach for advancing the study of CVDs and provides rich raw data for comprehensive investigation into cardiovascular health assessment and prediction models based on machine learning algorithms. Moreover, through extensive clinical comparative studies, it is anticipated that the system will find widespread application in evaluating cardiovascular health, drug efficacy, and postoperative rehabilitation.

## Electronic supplementary material

Below is the link to the electronic supplementary material.

Supplementary Material 1

Supplementary Material 2

Supplementary Material 3

Supplementary Material 4

Supplementary Material 5

Supplementary Material 6

Supplementary Material 7

Supplementary Material 8

Supplementary Material 9

Supplementary Material 10

Supplementary Material 11

Supplementary Material 12

Supplementary Material 13

## Data Availability

The data that support the findings of this study are not openly available due to reasons of sensitivity and are available from the corresponding author upon reasonable request.
