# Author Correction: Design and validation of a novel multiple sites signal acquisition and analysis system based on pressure stimulation for human cardiovascular information

**DOI:** 10.1038/s41598-025-08926-y

**Published:** 2025-07-02

**Authors:** Gaiqin Liu, Yuan Li, Longcong Chen, Juan Jiang, Jie Tian, Panpan Feng

**Affiliations:** 1 Medical Information College, Chongqing Medical University, Chongqing, People’s Republic of China; 2 Department of Cardiology, Children’s Hospital of Chongqing Medical University, Chongqing, People’s Republic of China; 3 Department of Cardiology, The First Affiliated Hospital of Chongqing Medical University, Chongqing, People’s Republic of China; 4 School of science, Chongqing University of Technology, Chongqing, People’s Republic of China; 5 Department of Geriatrics, Chongqing Mental Health Center, Chongqing, People’s Republic of China

Correction to: *Scientific Reports* 10.1038/s41598-025-97812-8, published online 18 April 2025

The original version of this Article contained an error in the name of the author Gaiqin Liu which was incorrectly given as Gaigin Liu.

The original Article has been corrected.

