# Supplementary material for: Design and validation of a novel multiple sites signal acquisition and analysis system based on pressure stimulation for human cardiovascular information
Source: Sci Rep. 2025 Apr 18;15:13392. doi: 10.1038/s41598-025-97812-8 (PMC12008263; doi:10.1038/s41598-025-97812-8)
Supplement: Supplementary file 2 — Supplementary Material 2 [file 41598_2025_97812_MOESM2_ESM.pdf]

Appendix A. Supplementary material

Fig. S2. The complete waveform of all 27-channel signals during the whole collection process about subject2

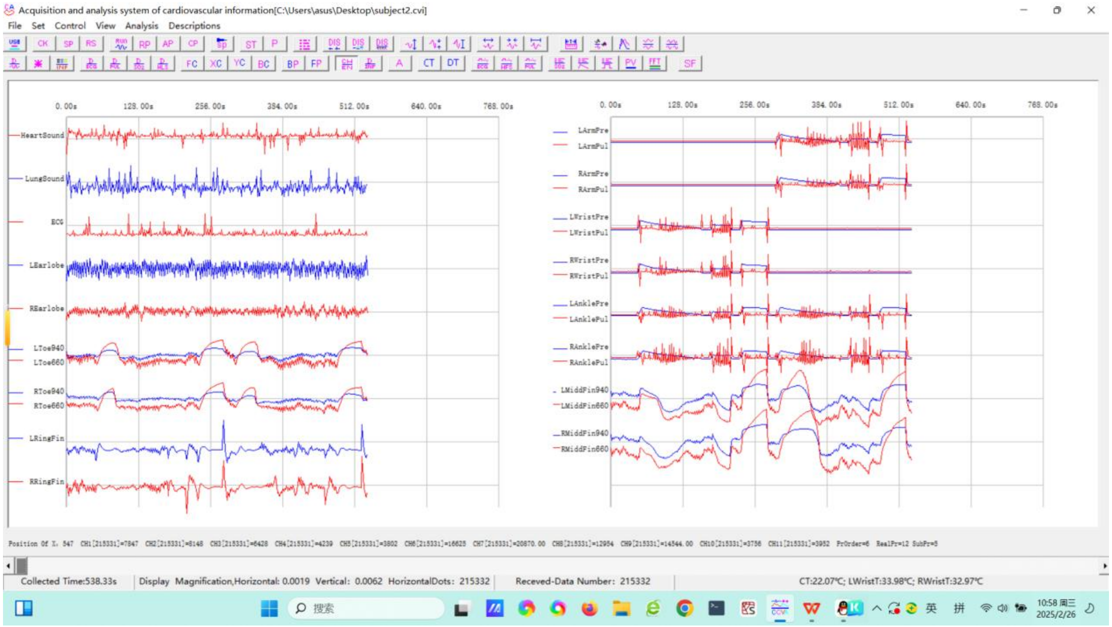

Fig. S2. The complete waveform of all 27-channel signals during the whole collection process about subject2
