# Supplementary material for: Design and validation of a novel multiple sites signal acquisition and analysis system based on pressure stimulation for human cardiovascular information
Source: Sci Rep. 2025 Apr 18;15:13392. doi: 10.1038/s41598-025-97812-8 (PMC12008263; doi:10.1038/s41598-025-97812-8)
Supplement: Supplementary file 3 — Supplementary Material 3 [file 41598_2025_97812_MOESM3_ESM.pdf]

Appendix A. Supplementary material

Fig. S3. Some parameters distribution about HS and LS

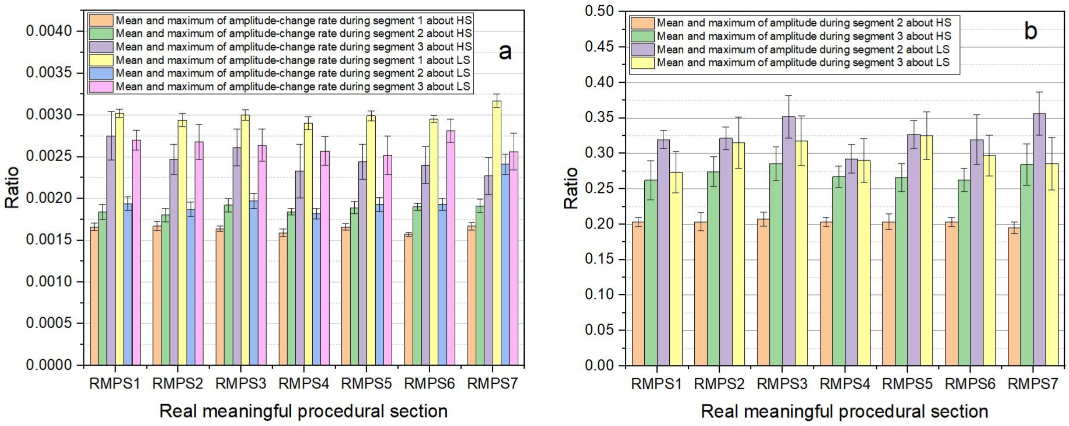

Fig. S3. Some parameters distribution about HS and LS. (a) The ratios of mean and maximum of amplitude-change rate during from segment 1 to 3 about HS and LS;(b) The ratios between mean and maximum of amplitude during from segment 1 to 3 about LS， the mean ratios of amplitude and amplitude-change rate of segment 2 to segment 1,and segment 3 to segment 1 about LS.
