# Supplementary material for: Design and validation of a novel multiple sites signal acquisition and analysis system based on pressure stimulation for human cardiovascular information
Source: Sci Rep. 2025 Apr 18;15:13392. doi: 10.1038/s41598-025-97812-8 (PMC12008263; doi:10.1038/s41598-025-97812-8)
Supplement: Supplementary file 4 — Supplementary Material 4 [file 41598_2025_97812_MOESM4_ESM.pdf]

Appendix A. Supplementary material

Fig. S4. Waveform diagram of measurement data from MS1 to MS8

Fig. S4.A. Partial waveform diagram of measurement data from MS1 to MS8

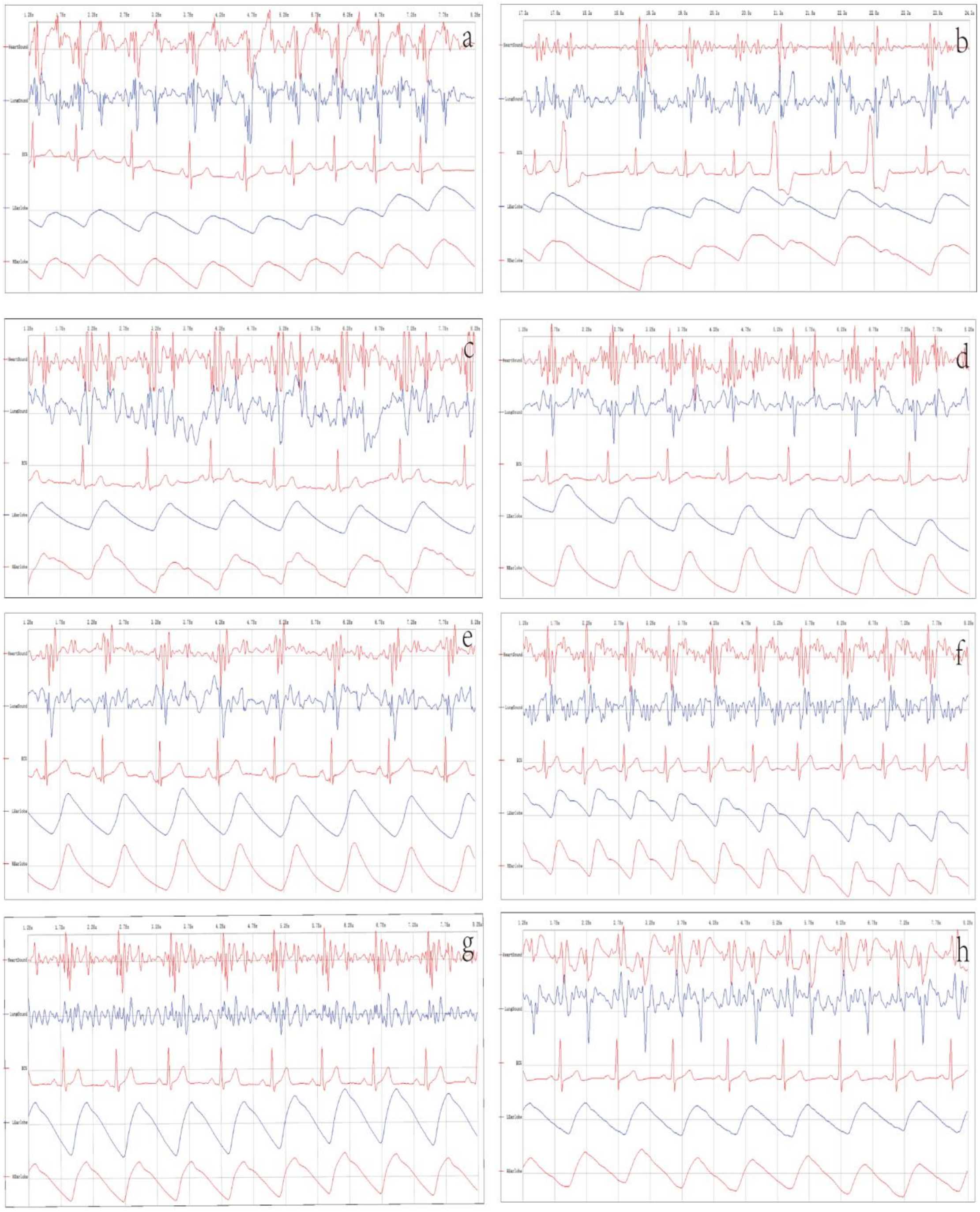

Fig. S4.A. Waveform of HS, LS, ECG and both-side OPSs at earlobes about the eight subjects. (a) about subject MS1; (b) about subject MS2; (c) about subject MS3; (d) about subject MS4 ; (e) about subject MS5; (f) about young subject MS6; (g) about middle-aged subject MS7 ;(h) about elderly subject MS8.

Fig. S4.B. Full waveform diagram of measurement data from MS1 to MS8

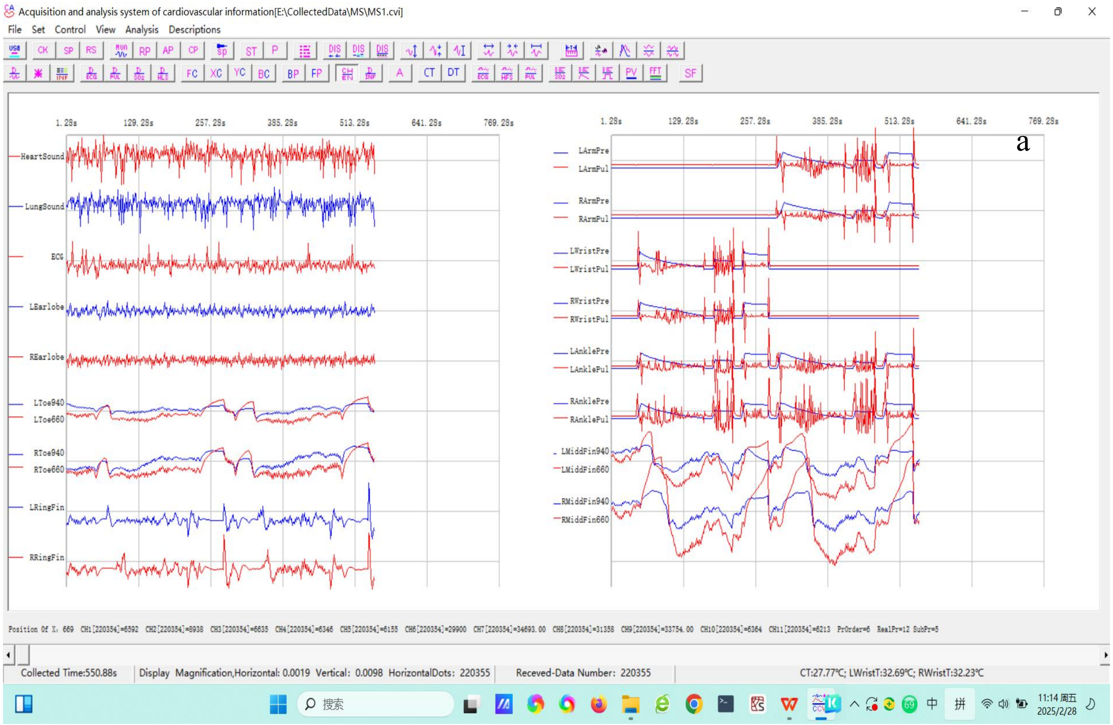

(a) Full waveform diagram of measurement data about MS1

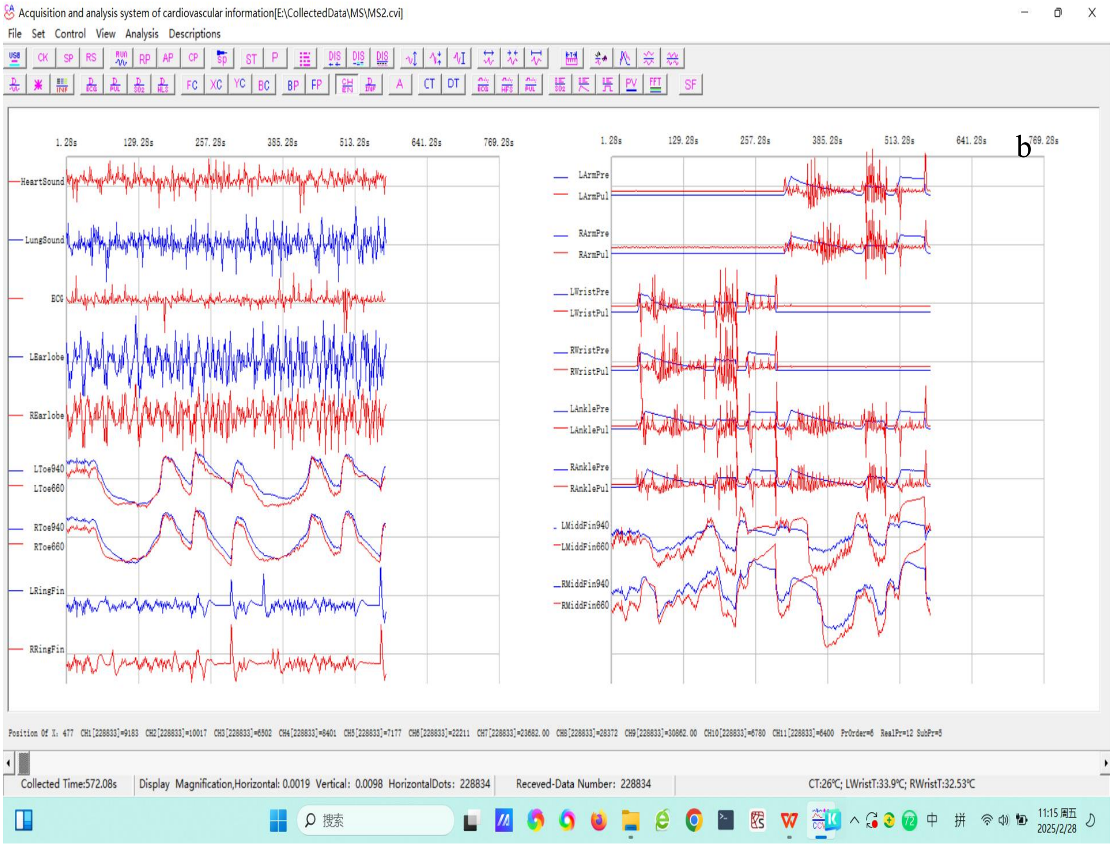

(b). Full waveform diagram of measurement data about MS2

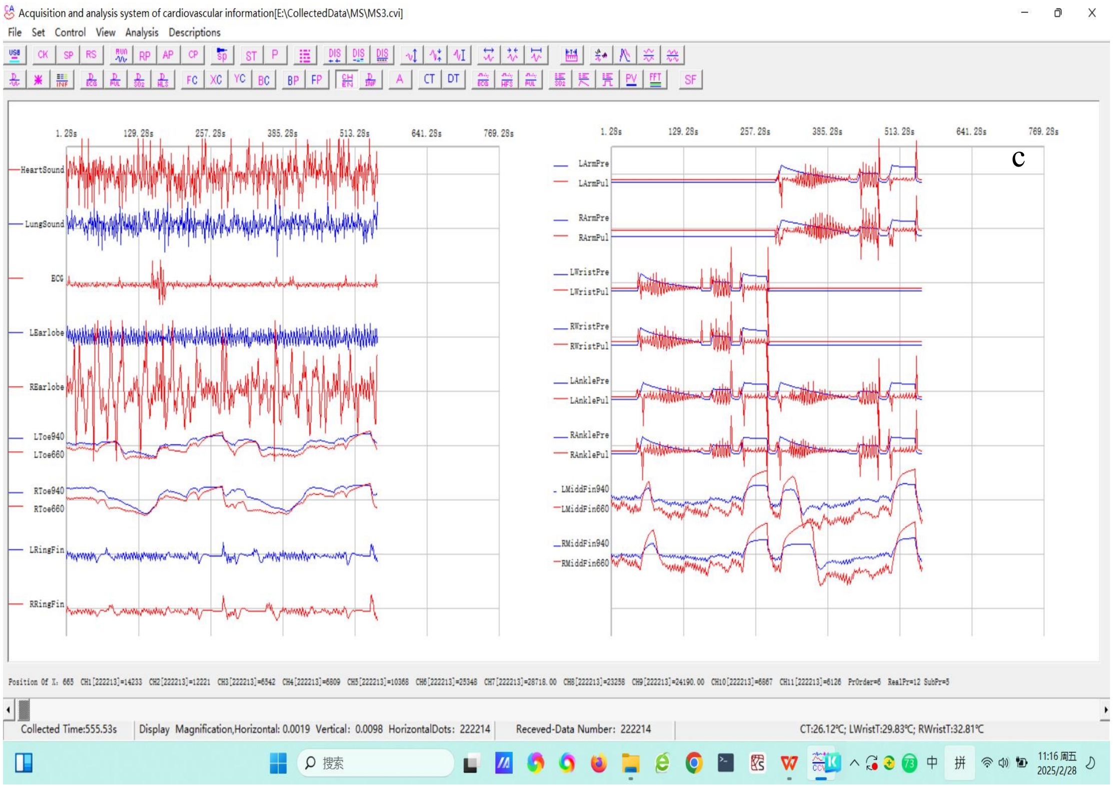

(c). Full waveform diagram of measurement data about MS3

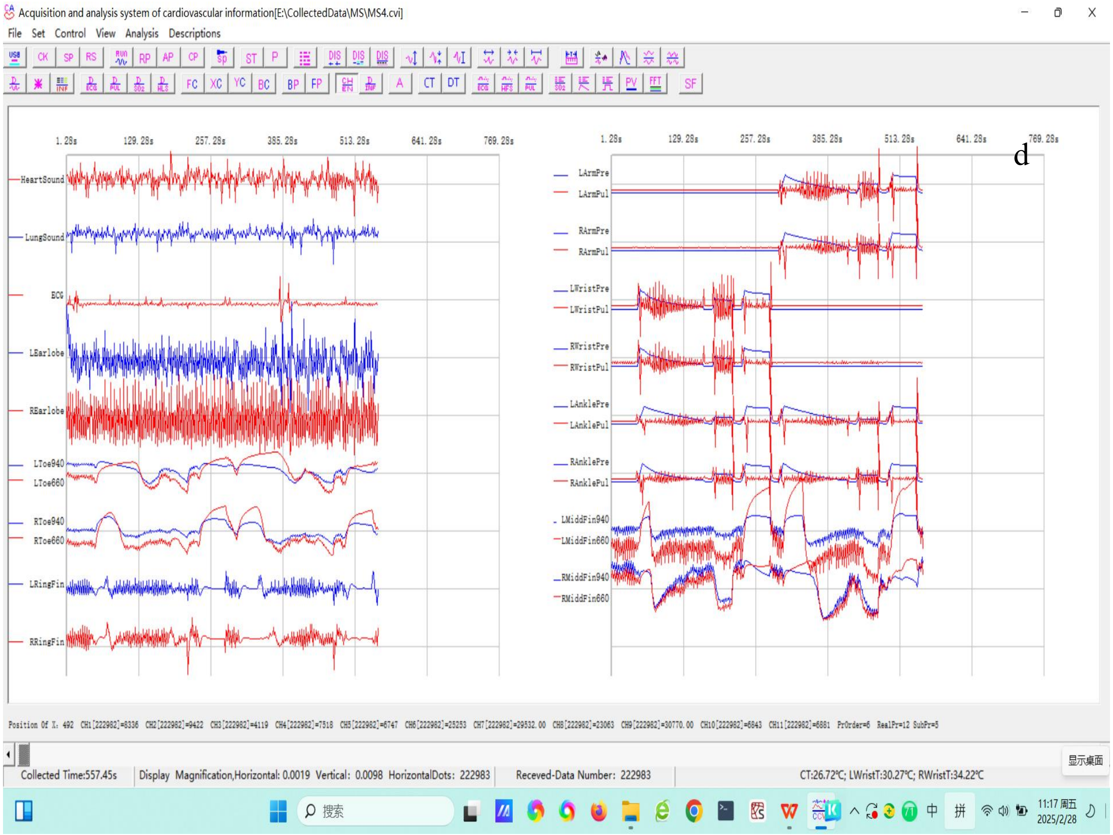

(d). Full waveform diagram of measurement data about MS4

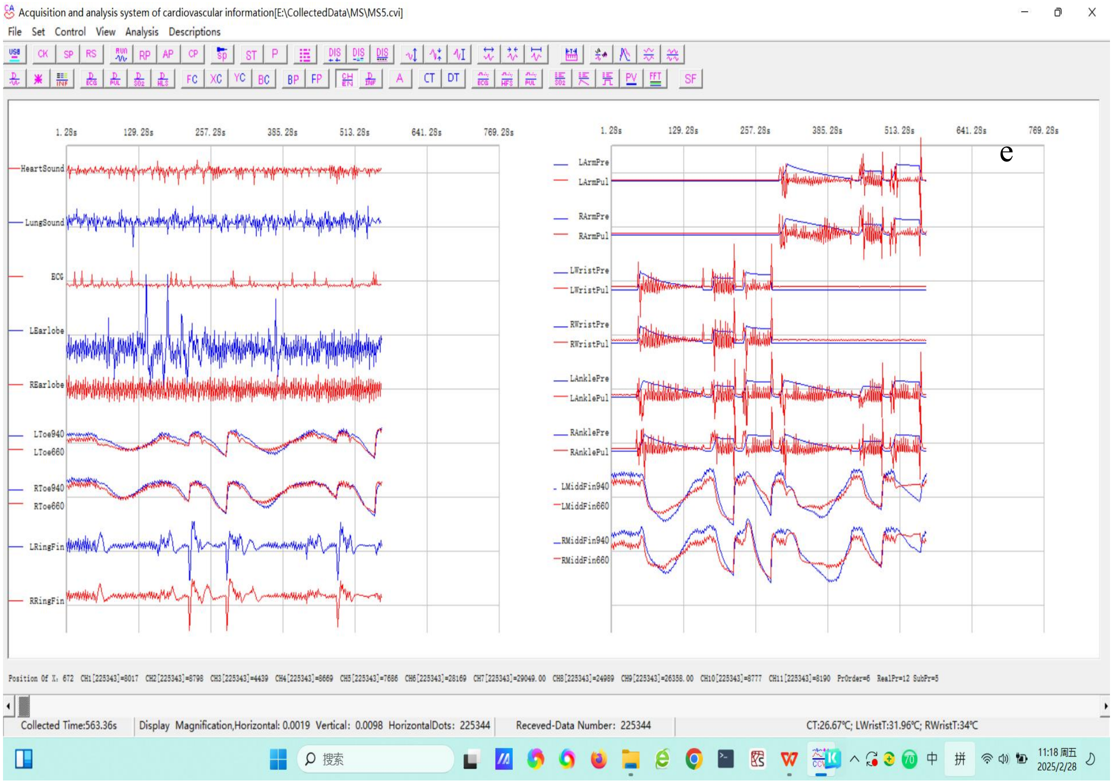

(e) Full waveform diagram of measurement data about MS5;

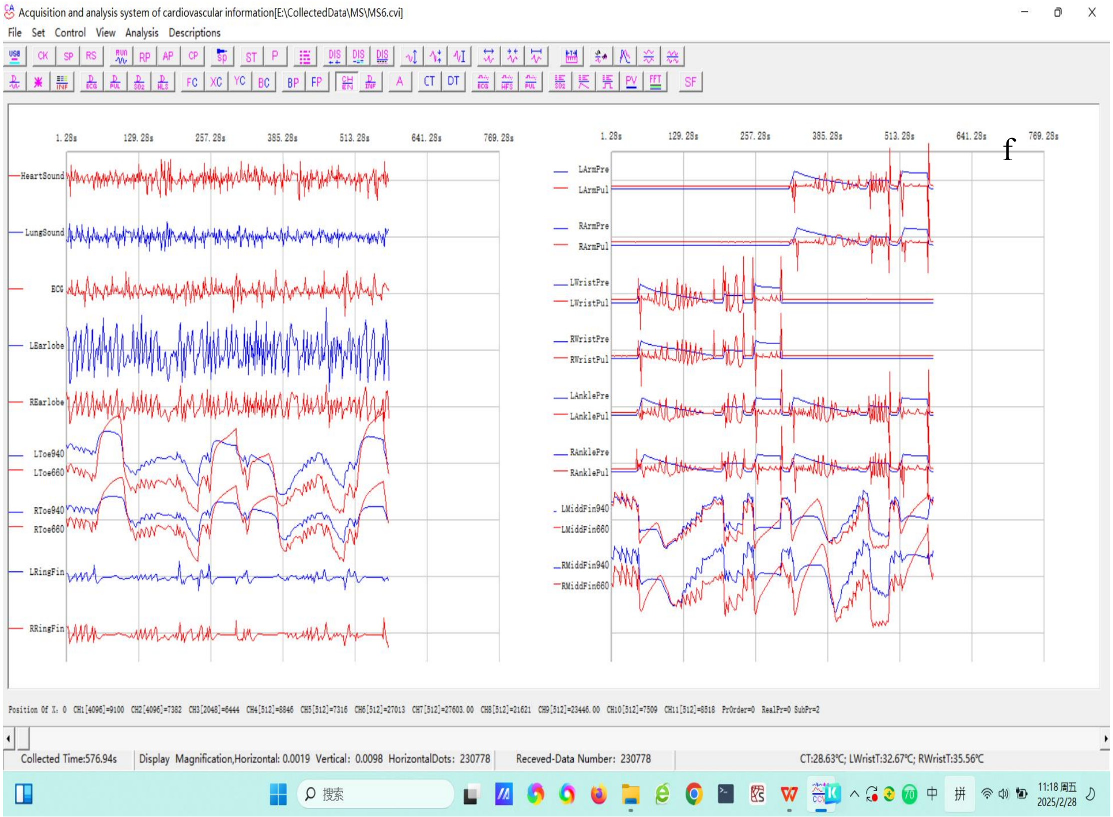

(f) Full waveform diagram of measurement data about MS6;

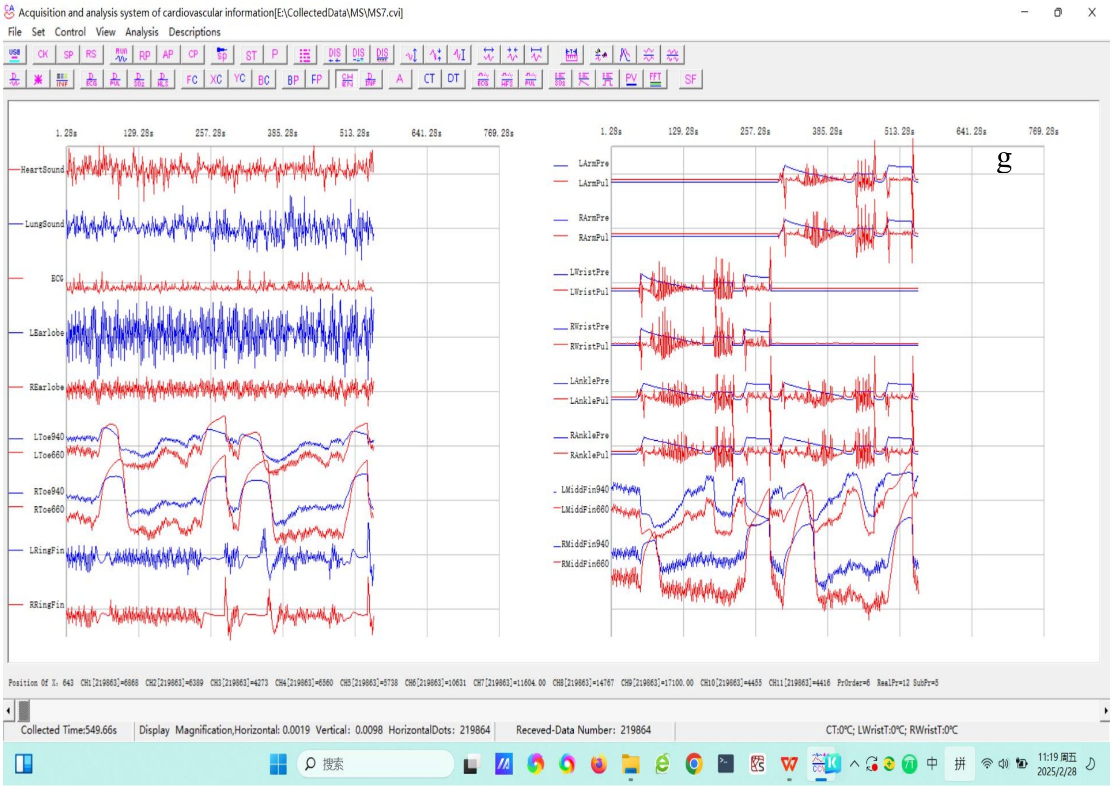

(g) Full waveform diagram of measurement data about MS7;

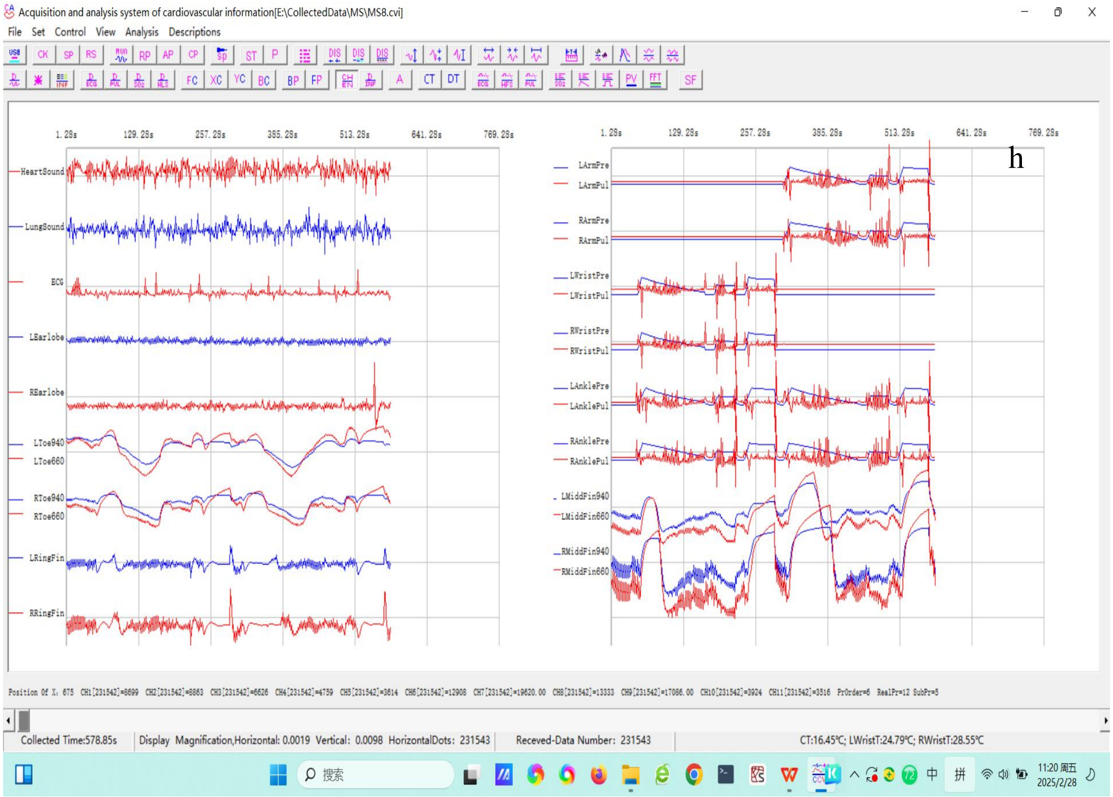

(h) Full waveform diagram of measurement data about MS8;

Fig. S4.B. Full waveform diagram of measurement data from MS1 to MS8.
