# Supplementary material for: Design and validation of a novel multiple sites signal acquisition and analysis system based on pressure stimulation for human cardiovascular information
Source: Sci Rep. 2025 Apr 18;15:13392. doi: 10.1038/s41598-025-97812-8 (PMC12008263; doi:10.1038/s41598-025-97812-8)
Supplement: Supplementary file 5 — Supplementary Material 5 [file 41598_2025_97812_MOESM5_ESM.pdf]

Appendix A. Supplementary material

Material S1. Amplitude distributions with frequency of 6-channel signals during RMSP1 about a subject

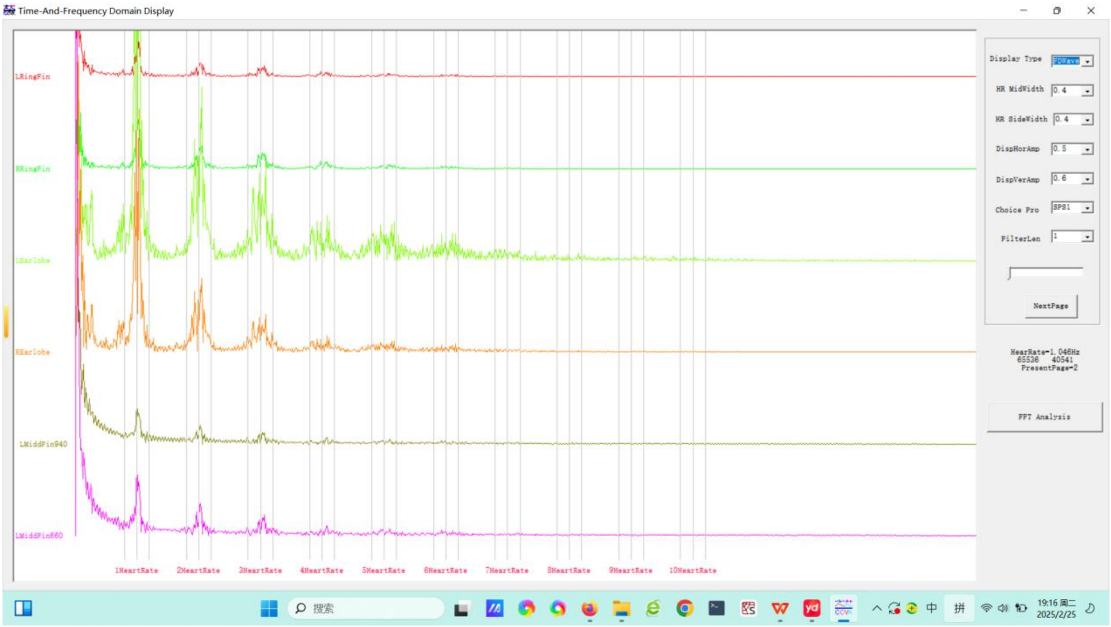

Fig. MS1.A. Amplitude distributions with frequency of 6-channel signals during RMSP1 about a subject

Additionally, For better understanding formula (9), its detailed explanation is as follows.

$$\mathrm{RAMF_{ijn}} = \frac{\mathrm{AveF_{ijn}}}{\mathrm{MaxF_{ijn}}} \tag{9}$$

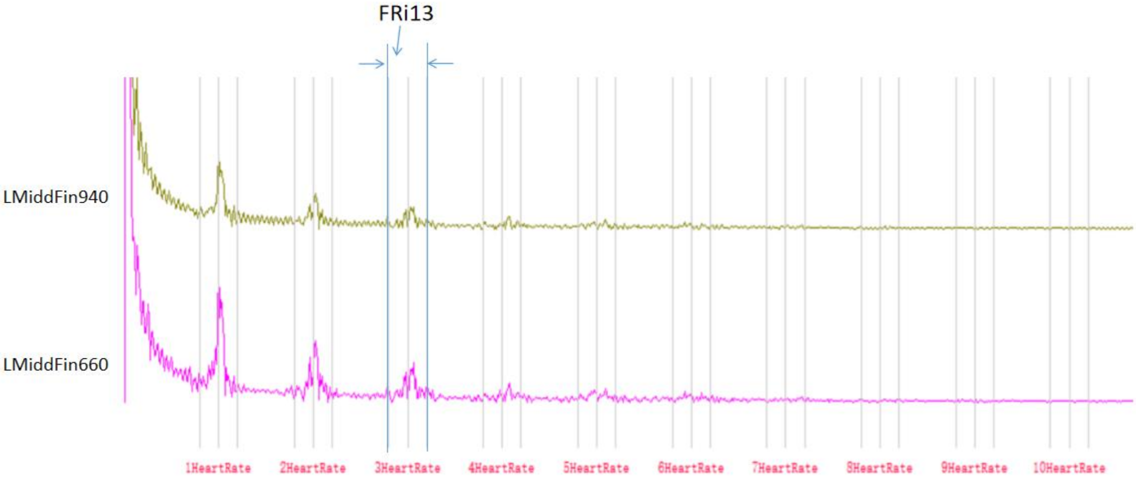

Fig. MS1.B. Amplitude distributions with frequency of 2-channel signals during RMSP1 about a

subject

In formula (11), i (range: 1-21) presents signal channel serial with the value range from 1 to 21, j (range: 1-7) is the RMPS serial, after performing FFT, n (range:1-10) expresses the serial of times heart rate (THR).

As Fig. MS1.B shown, $FR_{i13,}$ a frequency range, was featured as an example, $AverF_{i13}$ presents the average of amplitude during $FR_{i13,}$ $MaxF_{i13}$ is the maximum of amplitude during $FR_{i13,}$ Then the RAMFi13 can be computed by formula (9). Similarly, other $RAMF_{ijn}$ can be calculated.

From Fig. MS1.A, we can obtain that $RAMF_{ijn}$ is increasing as n increases.
