# Supplementary material for: Design and validation of a novel multiple sites signal acquisition and analysis system based on pressure stimulation for human cardiovascular information
Source: Sci Rep. 2025 Apr 18;15:13392. doi: 10.1038/s41598-025-97812-8 (PMC12008263; doi:10.1038/s41598-025-97812-8)
Supplement: Supplementary file 6 — Supplementary Material 6 [file 41598_2025_97812_MOESM6_ESM.pdf]

Appendix A. Supplementary material

Material S2. Exemplary results of Measuring 5 times about the subject2

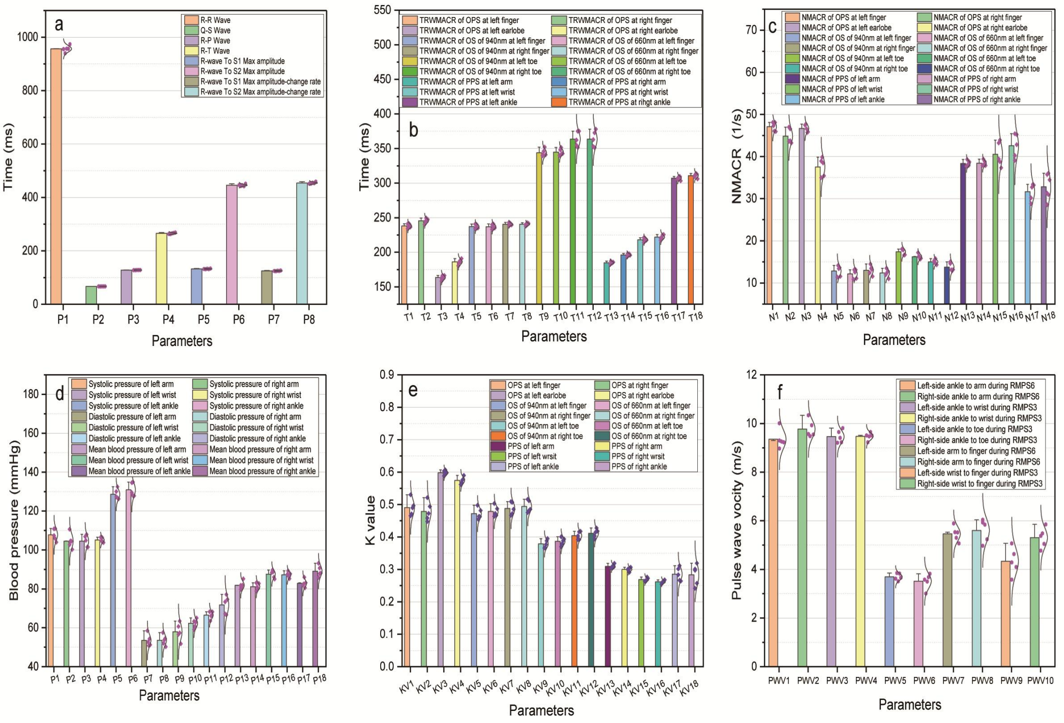

Fig. MS2. Distribution of some parameters about a subject for measuring five times. (a) The average time distribution of R-R wave, Q-S wave, R-P wave, R-T wave of ECG, and R-wave to maximum amplitude and maximum amplitude-change rate of S1 and S2 about HS;(b)The average time distribution of RWMACR of 18-channel signals;(c) The distribution of average NMACR of 18-channel signals;(d) The blood pressure-distribution of both-side wrists, arms and ankles;(e) The average K value distribution of 18-channel signals;(f) The PWV distribution of both-side ankles to arms, ankles to wrists, ankles to toes, arms to fingers, wrists to finger.

Particularly noteworthy is that the eight parameters (the average time of R-R wave, Q-S wave, R-P wave, R-T wave of ECG, and R-peak to maximum amplitude and maximum amplitude-change rate of S1 and S2 about HS), shown in Fig. S5(a), demonstrate excellent consistency across all five measurements conducted on the subject2. Furthermore, systolic and diastolic blood pressures measured using an electronic sphygmomanometer OMRON HEM-8102A at the subject's left arm were found to be 104 mmHg and 57 mmHg respectively, whereas when measured by our system on the subject's left arm for five times consecutively, they averaged at 107.8 mmHg and 53.48 mmHg respectively. The results obtained from both methods show a high degree of agreement. Moreover, the integration with

PPG, ECG, and other signals holds promise for enhancing blood pressure measurement accuracy. Overall, these findings indicate that our system exhibits good stability and provides reliable measurements.

Additionally, the subject was measured in the physical examination center of the First Affiliated Hospital of Chongqing Medical University by using the Omron arteriosclerotic instrument BP-203 RPEIII, and the tested results of this instrument and our designed system are listed in Table 4. The results show that the measured systolic blood pressure of the left arm has the largest difference of 6.80 mmHg, but the rest of the difference is generally within the normal range (less than 5 mmHg). Therefore, on the whole, the results of this system are consistent with those measured by this instrument, which means that the blood pressure measurement of this system exhibits a certain level of reliability. Certainly, by further optimizing the calculation coefficient of systolic and diastolic blood pressure, the measurement accuracy of blood pressure can be improved.

Table MS2. Blood pressure parameters.

Pressure Type	Left Arm	Right Arm	Left wrist	Right wrist	Left ankle	Right ankle
Diastolic Pressure	53.48	53.58	57.97	62.25	66.49	71.75
Diastolic Pressure*	58	58	-	-	68	70
Mean pressure	81.86	81.14	87.60	87.19	82.82	89.12
Mean pressure*	77	77	-	-	81	88
Systolic Pressure	107.80	104.53	104.55	105.09	128.57	130.92
Systolic Pressure*	101	107	-	-	126	129
Pulse pressure	54.32	50.95	46.58	42.84	62.09	59.18
Pulse pressure*	43	49	-	-	58	59

Note: * indicates the result measured by the Omron arteriosclerotic instrument BP-203 RPEIII
