# Supplementary material for: Design and validation of a novel multiple sites signal acquisition and analysis system based on pressure stimulation for human cardiovascular information
Source: Sci Rep. 2025 Apr 18;15:13392. doi: 10.1038/s41598-025-97812-8 (PMC12008263; doi:10.1038/s41598-025-97812-8)
Supplement: Supplementary file 7 — Supplementary Material 7 [file 41598_2025_97812_MOESM7_ESM.pdf]

Appendix A. Supplementary material

Method S1. The method of obtaining the calculated cycles

Compute the data of 5-point smoothing filter about ECG,

$$\mathrm{FD}[m] = \frac{1}{5}\sum_{k=-2}^{+2}\mathrm{D}[m+k] \qquad \text{(S1)}$$

Where m denotes the order of data in sequences, k represents a variable whose value is from -2 to +2. D[m+k] refers to the data sequence of output value about ECG, FD[n] presents the data sequence of ECG after applying five-dot smoothing filter.

Then, calculate the difference data sequence, DFD[m],

$$\mathrm{DFD}[\mathrm{m}] = \mathrm{FD}[m+1] - \mathrm{DF}[m] \qquad \text{(S2)}$$

If MaxDFD and MinDFD respectively represent the maximum and minimum value of data sequence DFD[m] when m changes from 1000 to 6000, the upper and lower thresholds, recorded as UpTH and LoTH severally, can be obtained by the following formulas (S3) and (S4) for searching R-peak positions of ECG.

$$\text{UpTH= MaxDFD×0.5} \qquad \text{(S3)}$$

$$\text{LoTH= MinDFD×0.5} \qquad \text{(S4)}$$

To mitigate the impact of initial part data, R-peak positions of ECG are searched from m=800 to the end in DFD[m]. Meanwhile, its the number, marked as p , is initialized to zero. Then the valid R-peak positions of ECG can be acquired through following a series of specific steps.

(a) Add 1 to m continually until DFD [m] is bigger than UpTH, and mark current value of m as StPos.

(b) Keep Adding 1 to m ceaselessly until DFD [m] is smaller than LoTH, and recorder current value of m as EnPos.

(c) From m = StPos to EnPos, search the position of maximum value of FD[m], namely R-peak position of ECG, denoted as RWPos. Set RPos[p] as the data sequence of RWPsECG, then RPos[p]=RWPos, and p is incremented by 1.

(d) Repeat step (a)-(c), find out each position of R-peak positions of ECG in turn, until the last data of DFD [m] is searched.

(e) Set data sequence DRPos [p] = RPos [p + 1] - RPos [p] , and arrange them from largest to smallest, then calculate the average of the middle fifty percent data (MFPD), marked as AverDRPos.

(f) If value of DRPos [p] is between AverDRPos×1.3 and AverDRPos×0.7, RPos [p + 1] and RPos [p] are both recorded as valid R-peak positions of ECG, while the others as invalid. And only the time between each adjacent two valid R-peak positions of ECG during the RMPSs is regarded as a calculated cycle, as shown in Fig. MS1.

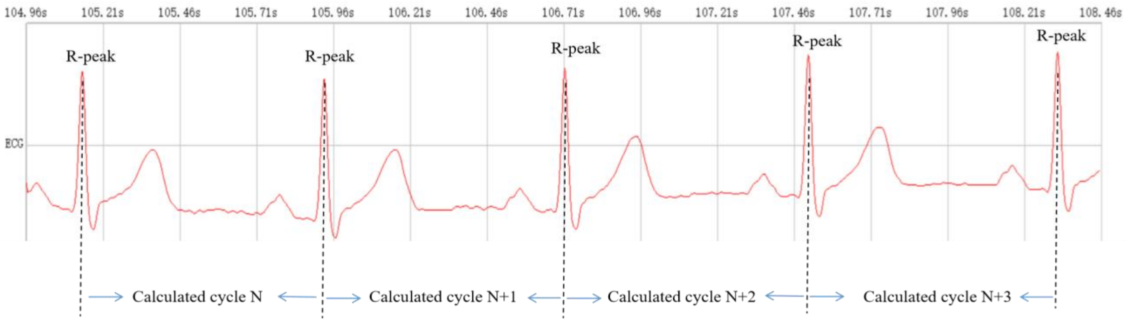

Fig. MS1. Five R-peak positions of ECG and four calculated cycles.
