# Supplementary material for: Design and validation of a novel multiple sites signal acquisition and analysis system based on pressure stimulation for human cardiovascular information
Source: Sci Rep. 2025 Apr 18;15:13392. doi: 10.1038/s41598-025-97812-8 (PMC12008263; doi:10.1038/s41598-025-97812-8)
Supplement: Supplementary file 8 — Supplementary Material 8 [file 41598_2025_97812_MOESM8_ESM.pdf]

Appendix A. Supplementary material

Method S2. The Details of respective parameters about the 18-channel signals

Based on the ECC, compute the parameters of 18-channel signals, some of which are displayed in Fig. MS2. Such as the difference (A_d) between maximum and minimum value (DMNV), NMCR, K Value calculated by formula (MS1), the time from R-wave of ECG to positions of maximum value, minimum value and maximum-change rate during the ECC (t_{RMAX}, t_{RMin}, t_{RDMAX}, t_{RFMin}), and their average, standard deviation, normalized standard deviation (NSD) calculated by formula (MS2), some ratio during the some RMPSs ,etc..

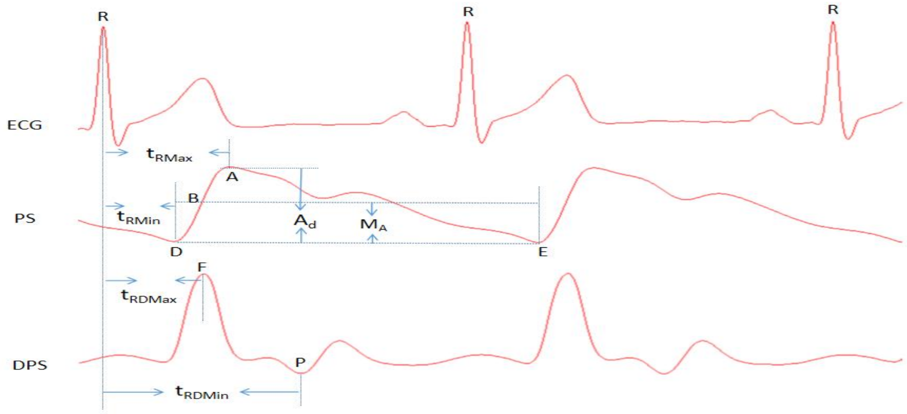

Fig. MS2. Schematic diagram of some characteristic parameters of pulse signals. PS expresses the pulse signal,which is PPG or PPS, DPS presents differential signal of PS.

$$\text{K value} = \frac{\text{Md}}{\text{Ad}} \qquad \text{(MS1)}$$

$$\text{NSD} = \frac{\text{SD}}{\text{MAd}} \qquad \text{(MS2)}$$

Where Md is the mean value of the alternating component of calculated signal during ECC, Ad expresses the difference between maximum and minimum value of the calculated signal during ECC, SD is standard deviation of calculated parameter during given RMPS, MAd presents the mean of differences between maximum and minimum value of all calculated cycles during given RMPS, NSD is normalized standard deviation.
