# Supplementary material for: Design and validation of a novel multiple sites signal acquisition and analysis system based on pressure stimulation for human cardiovascular information
Source: Sci Rep. 2025 Apr 18;15:13392. doi: 10.1038/s41598-025-97812-8 (PMC12008263; doi:10.1038/s41598-025-97812-8)
Supplement: Supplementary file 9 — Supplementary Material 9 [file 41598_2025_97812_MOESM9_ESM.pdf]

Appendix A. Supplementary material

Table S1. Abbreviation and their full names in this article

Serial Number	Abbreviation	Full words
1	CVDs	Cardiovascular diseases
2	CVI	Cardiovascular information
3	CVPs	Cardiovascular parameters
4	PWV	Pulse wave velocity
5	ECG	Electrocardiogram
6	HS	Heart sound
7	PPG	Photoplethysmography
8	MPAP	Maximum pulse amplitude pressure
9	BBFP	blocking blood flow pressure
10	LS	Lung sound
11	USB	Universal serial bus
12	PPGSs	Photoplethysmography signals
13	HLSSs	Heart and lung sound signals
14	PPPSs	Pressure and pressure pulse signals
15	BODWSs	Blood-oxygen double wavelength signals
16	CDA	Charge-and-discharge air
17	DC-DC	current to direct current
18	RMPSs	Real meaningful procedural segments
19	MP	Maximum pressure;
20	MPAP	Maximum pulse amplitude pressure.
21	SPO2	Saturation of peripheral oxygen
22	ADCV	Analog-digital conversion value
23	ECC	Each calculated cycle
24	NMACR	Normalized maximum amplitude-change rate
25	NSD	Normalized standard deviation
26	NSDs	Normalized standard deviations
27	OS	Optoelectronic signal
28	HR	Heart rate
29	THR	Times heart rate
30	FFT	Fast Fourier transform
31	RWMACR	R-wave to maximum amplitude-change rate
32	OSs	Optoelectronic signals
33	OPSs	Optoelectronic pulse signals
34	PPSs	pressure pulse signals
35	NMARR	Normalized maximum-amplitude rise rate
