# Supplementary material for: Design and validation of a novel multiple sites signal acquisition and analysis system based on pressure stimulation for human cardiovascular information
Source: Sci Rep. 2025 Apr 18;15:13392. doi: 10.1038/s41598-025-97812-8 (PMC12008263; doi:10.1038/s41598-025-97812-8)
Supplement: Supplementary file 10 — Supplementary Material 10 [file 41598_2025_97812_MOESM10_ESM.pdf]

Appendix A. Supplementary material

Table S2. The table of parameters between pressure and ADCV about each pressure signal

Table S2. The parameters between pressure and ADCV about each pressure signal

Cuffs		Intercept Value	Intercept Standard Error	Slope Value	Slope Standard Error	Adj. R-Square	Error of Mean Square
Left	arm	-18.59	0.060	0.018	7.03E-6	1.00000	0.0068
Right	arm	-21.09	0.081	0.016	8.50E-6	1.00000	0.0122
Left	wrist	-16.30	0.114	0.019	1.48E-5	0.99999	0.0253
Right	wrist	-18.27	0.094	0.018	1.10E-5	1.00000	0.0166
Left	ankle	-21.01	0.083	0.018	9.77E-6	1.00000	0.0128
Right	ankle	-28.44	0.094	0.016	9.39E-6	1.00000	0.0150
