# Supplementary material for: Design and validation of a novel multiple sites signal acquisition and analysis system based on pressure stimulation for human cardiovascular information
Source: Sci Rep. 2025 Apr 18;15:13392. doi: 10.1038/s41598-025-97812-8 (PMC12008263; doi:10.1038/s41598-025-97812-8)
Supplement: Supplementary file 11 — Supplementary Material 11 [file 41598_2025_97812_MOESM11_ESM.pdf]

Appendix A. Supplementary material

Table S3. The fitting coefficients of systolic and diastolic pressure about each pressure signal

Table S3. The fitting coefficients of systolic and diastolic pressure about each pressure signal

The channel of pressure signal	Fitting coefficient of systolic pressure	Standard deviation	Fitting coefficient of systolic pressure	Standard deviation
Left arm	0.56	0.027	0.55	0.014
Right arm	0.54	0.030	0.58	0.018
Left wrist	0.56	0.021	0.54	0.017
Right wrist	0.55	0.025	0.55	0.016
Left ankle	0.55	0.028	0.56	0.016
Right ankle	0.55	0.030	0.55	0.014
