# Supplementary material for: Design and validation of a novel multiple sites signal acquisition and analysis system based on pressure stimulation for human cardiovascular information
Source: Sci Rep. 2025 Apr 18;15:13392. doi: 10.1038/s41598-025-97812-8 (PMC12008263; doi:10.1038/s41598-025-97812-8)
Supplement: Supplementary file 12 — Supplementary Material 12 [file 41598_2025_97812_MOESM12_ESM.pdf]

Appendix A. Supplementary material

Table S4. Basic information of sixteen volunteers (MS1-MS8 and Subject1-Subject8)

Table S4(a). Basic information of 8 volunteers (MS1-MS8)

Item	MS1	MS2	MS3	MS4	MS5	MS6	MS7	MS8	Mean±SD
Age (years)	21	73	55	77	59	20	50	69	53±22
Gender	female	male	male	female	female	male	male	female	-
Height (cm)	159	170	160	159	160	178	168	152	163.3±8.2
Weight (Kg)	49.4	71.5	58.0	57.0	67.5	68.8	66.4	48.7	60.9±8.9
Heart Rate (bpm)	84	87	61	66	68	91	72	84	76.6±11.2
Systolic BP (mmHg)	117	137	106	112	153	117	113	129	123.0±15.7
Diastolic BP (mmHg)	82	89	67	57	82	72	68	55	71.5±12.2
Work Type	mental labor	physical labor	physical labor	mental labor	physical labor	mental labor	mental labor	physical labor	-
Diabetes	not	not	not	Type II	Type II	not	not	not	-
Hypertension	not	yes	not	yes	yes	not	not	not	-
Physical Exercises	few	often	often	few	few	few	few	often	-
Other known information	arrhyt-hmia	Arrhythmia, premature beat, Bilateral carotid intimedia thickened	Right internal carotid artery stenosis, stenosis rate about 50-70%	left carotid stenosis, right subclavian atherosclerotic plaque formation; Atherosclerotic plaque formation in both lower limbs, Peripheral atherosclerosis	Bilateral carotid atherosclerotic plaque formation; Atherosclerotic plaque in the right subclavian artery Peripheral atherosclerosis	Increased right vertebral artery flow resistance	No CVDs	No CVDs	-

Note: CVDs---cardiovascular diseases.

Table S4(b). Basic information of other 8 volunteers (Subject1-Sbuject8)

Serial Number	9	15	10	11	12	13	14	16
Name identification	Subject1	Subject2	Subject3	Subject4	Subject5	Subject6	Subject7	Subject8
Gender	male	male	male	female	female	male	female	female
Age	24	24	23	23	23	49	57	22
Height (cm)	176	171	165	174	158	169	157	160
Weight (Kg)	71.5	65.4	74.4	64	52.8	70	62	49
Systolic Pressure (mmHg)	131	110	111	111	112	141	108	108
Diastolic Pressure (mmHg)	78	58	76	80	67	99	81	73
Distances between wrist and middle finger (mm)	200	180	170	185	174	188	175	180
Distances between arm and wrist (mm)	390	365	320	340	330	335	316	318
Distances between ankle and middle tiptoe (mm)	200	190	160	180	178	198	190	172
Work Type								
Occupation	student	student	student	student	student	salesman	peasant	student
Heart rate	65	95	96	85	83	72	90	67
Diabetes	not	not	not	not	not	not	not	not
Hypertension	not	not	not	not	not	yes	yes	not
Physical Exercises	few	few	few	few	often	few	few	few
Other known information	No CVDs	No CVDs	No CVDs	No CVDs	No CVDs	No cCVDs	No CVDs	No CVDs

Note: CVDs---cardiovascular diseases.
