# Supplementary material for: Design and validation of a novel multiple sites signal acquisition and analysis system based on pressure stimulation for human cardiovascular information
Source: Sci Rep. 2025 Apr 18;15:13392. doi: 10.1038/s41598-025-97812-8 (PMC12008263; doi:10.1038/s41598-025-97812-8)
Supplement: Supplementary file 13 — Supplementary Material 13 [file 41598_2025_97812_MOESM13_ESM.pdf]

Appendix A. Supplementary material

Table S5. The abbreviation and their full names in our designed system software

Table S5. The relationships between abbreviated letters and their means

The abbreviated letters	its means
HeartSound	Heart sound signal
LungSound	Lung sound signal
ECG	Electrocardiogram signal
LMiddleFin	Optoelectronic pulse signal of 940 nm at left middle finger
RMiddleFin,	Optoelectronic pulse signal of 940 nm at right middle finger
LEarlobe	Optoelectronic pulse signal of 940 nm at left earlobe
REarlobe	Optoelectronic pulse signal of 940 nm at right earlobe
LIndexFin940	Optoelectronic signal of 940 nm at left index finger
LIndexFin660	Optoelectronic signal of 660 nm at left index finger
RIndexFin940	Optoelectronic signal of 940 nm at right index finger
RIndexFin660	Optoelectronic signal of 660 nm at right index finger
LToe940	Optoelectronic signal of 940 nm at left middle toe
LToe660	Optoelectronic signal of 660 nm at left middle toe
RToe940	Optoelectronic signal of 940 nm at right middle toe
RToe660	Optoelectronic signal of 660 nm at right middle toe
LArmPre	Pressure signal at left arm
LArmPul	Pressure pulse signal at left arm
RArmPre	Pressure signal at right arm
RArmPul	Pressure pulse signal at right arm
LWristPre	Pressure signal at left wrist
LWristPul	Pressure pulse signal at left wrist
RWristPre	Pressure signal at right wrist
RWristPul	Pressure pulse signal at right wrist
LAnklePre	Pressure signal at left ankle
LAnklePul	Pressure pulse signal at left ankle
RAnklePre	Pressure signal at right ankle
RAnklePul	Pressure pulse signal at right ankle
